# Computationally Guided Design and Experimental Validation of ssDNA Aptamers Targeting the SARS-CoV-2 Spike Protein

**DOI:** 10.3390/biom16101476

**Published:** 2026-10-10

**Authors:** José C. E. Márquez Montesinos, Ana Maria Bernardez, Yuliet Mazola, Lucianna Vaccaro, Yanira Sáez-Álvarez, Rubén Agudo, Elizabeth Valdivieso, Sergio Llorens-Berzosa, Soledad Fenoy, Wendy González, Fernando Izquierdo, Dolores Ollero, Carmen del Aguila, Carolina Hurtado-Marcos

**Affiliations:** 1 Centro de Bioinformática, Simulación y Modelado (CBSM), Universidad de Talca, Talca, Chile; jose.marquez@utalca.cl (J.C.E.M.M.); yuliet.mazola@utalca.cl (Y.M.); wgonzalez@utalca.cl (W.G.); 2 Centro de Nanomedicina, Diagnóstico y Desarrollo de Fármacos (ND3), Laboratorio de Fisiología Molecular, Escuela de Medicina, Universidad de Talca, Talca, Chile; 3 Facultad de Farmacia, Universidad San Pablo-CEU, CEU Universities, Urbanización Montepríncipe, 28660 Boadilla del Monte, Spain; ana.bernardezsanchez@ceu.es (A.M.B.); lucianna.vaccaro@ceu.es (L.V.); yanira.saezalvarez@ceu.es (Y.S.-Á.); ruben.agudotorres@ceu.es (R.A.); elizabeth.valdiviesoblanco@ceu.es (E.V.); scultivos@ceu.es (S.L.-B.); sfenrod@ceu.es (S.F.); ferizqui@ceu.es (F.I.); mariadolores.ollerobaceiredo@ceu.es (D.O.); cagupue@ceu.es (C.d.A.)

**Keywords:** aptamers, computational design, molecular docking, SARS-CoV-2 Spike protein, biosensing, analytical diagnostics, in silico pipeline

## Abstract

Aptamer development has traditionally relied on iterative, labor-intensive experimental approaches. Recent advances in bioinformatics and computational power now enable the rational in silico design of high-affinity nucleic acid ligands, drastically reducing time and cost. Here, we present an integrated computational pipeline for designing ssDNA aptamers targeting the receptor-binding domain (RBD) of the SARS-CoV-2 Spike (S) protein. A library of 4000 randomly generated 20-nucleotide sequences with defined base ratios (C:G:A:T = 3:3:2:2) was screened based on secondary structure and free energy. The 100 most stable sequences (ΔG ≤ −4.1 kcal/mol) were modeled in three dimensions, yielding 1000 conformations. The 100 structures with the lowest potential energies (≤−78,051.7 kcal/mol) were selected for molecular docking with the RBD in the closed, open, and transitional states. Two aptamers—model-2-seq_2714 (seq_2714) and model-5-seq_1397 (seq_1397)—were selected for experimental assessment based on their favorable predicted docking scores against the RBD. Western blot and dot blot assays showed detectable recognition of the recombinant RBD by both aptamers under the experimental conditions tested. Exploratory testing in a limited number of clinical and environmental samples further showed that detectable signals could be obtained in selected complex matrices. These findings provide an initial proof-of-principle assessment of a computational-first strategy for prioritizing candidate aptamers for subsequent experimental testing.

## 1. Introduction

Aptamers are synthetic nucleic acid molecules (DNA or RNA) that have the ability to bind with high affinity and specificity to a wide variety of biological targets, such as proteins, cells, viruses, or small molecules. This molecular recognition ability is due to their specific three-dimensional structure, which allows them to mimic the mode of action of antibodies, albeit with some notable advantages. Unlike antibodies, aptamers can be generated against smaller molecules, exhibit greater stability under adverse environmental conditions (they are stable at room temperature), are more cost-effective to produce, and can be chemically modified with relative ease. These characteristics make them highly promising tools for applications in diagnostics, targeted therapy, controlled drug delivery, and biosensors [1,2].

Recent global health challenges have underscored the importance of developing robust, rapid, and adaptable diagnostic systems. Among emerging molecular recognition tools, aptamers have gained considerable attention due to their synthetic accessibility, stability, and tunable binding properties. The extensive characterization of SARS-CoV-2 and its structural proteins provides a valuable framework for validating aptamer-based detection strategies, positioning this virus as an optimal model for advancing next-generation diagnostic platforms. One of the main therapeutic and diagnostic targets of this virus has been the S protein, located on the surface of the virus, which facilitates viral entry into human cells through interaction with the angiotensin-converting enzyme 2 (ACE2) receptor. Given the importance of this protein in the viral life cycle, its early detection or inhibition is considered a strategic goal to curb the spread of the virus. In this regard, aptamers specifically designed to recognize the S protein represent an effective and complementary alternative to monoclonal antibodies, particularly in situations where speed, stability, and low production costs are critical [3,4].

Several studies have already reported aptamers targeting the RBD of the SARS-CoV-2 S protein, demonstrating their applicability in viral detection and neutralization assays [2,3,5,6,7,8].

More recent studies have further advanced the development and characterization of DNA aptamers targeting SARS-CoV-2 S proteins. Auti et al. (2025) developed novel ssDNA aptamers against the RBD using SELEX, followed by experimental binding assessment and computational docking analyses [8]. In parallel, Poolsup et al. (2025) reported DNA aptamers targeting the S1 subunit, including the Omicron variant, with nanomolar binding affinities and detailed epitope characterization [7]. These studies highlight the continued development of experimentally selected aptamers against SARS-CoV-2 S proteins and emphasize the value of integrating computational and experimental approaches for aptamer characterization [7,8].

Traditionally, the selection of aptamers has been carried out through the SELEX method (Systematic Evolution of Ligands by EXponential Enrichment), an iterative process that progressively enriches an initial library of random sequences to obtain those with the highest affinity for a given target [9,10]. Although robust, this method typically requires multiple rounds of selection and amplification. Variants such as CE-SELEX, which incorporate capillary electrophoresis techniques, have improved the efficiency and reduced the number of necessary cycles, thereby increasing the precision in separating active sequences [11].

**Figure 1:**
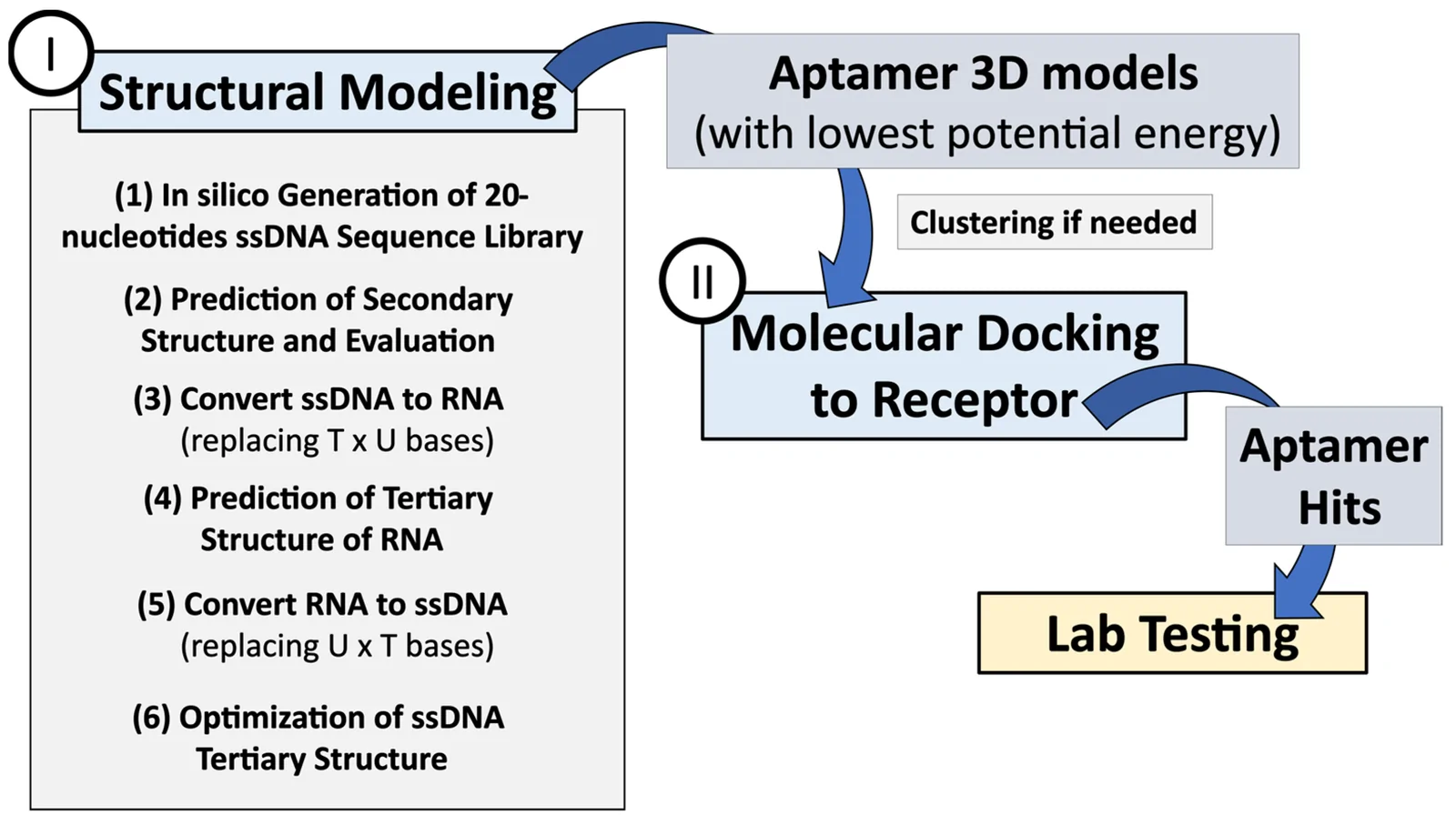
Computational aptamer design workflow. Schematic overview of the computational pipeline used for aptamer discovery. Candidate sequences were generated and subjected to (I) structural modeling, energetic stability filtering and (II) molecular docking to predict high-affinity interactions with the target protein. The most promising candidates identified in silico were subsequently selected for experimental assessment by Western blot and dot blot assays.

The rapid evolution of computational biology has fundamentally redefined the conceptual framework of aptamer discovery. While traditional experimental selection methods such as SELEX remain powerful, they are inherently time-consuming, resource-intensive, and limited in their ability to exhaustively explore the vast combinatorial sequence space of nucleic acids. Recent advances in computational approaches for aptamer discovery have further expanded the capabilities of rational aptamer engineering [12]. In contrast, contemporary in silico methodologies enable rational, structure-guided design strategies capable of interrogating conformational landscapes, thermodynamic stability, and binding energetics at near-atomic resolution. Although computational design methods are increasingly used to guide aptamer selection, fully integrated in silico-first workflows followed by experimental assessment of computationally prioritized candidates are still comparatively limited in the literature [3,12,13]. By integrating single-strain DNA (ssDNA) secondary structural prediction (Figure 1), ssDNA tridimensional modeling and molecular docking, computational pipelines allow for the systematic pre-screening and optimization of candidate sequences prior to synthesis. This shift from empirical selection toward rational structural engineering represents a true paradigm change in aptamer development, enhancing efficiency, reproducibility, scalability, and mechanistic interpretability. Rather than serving as a complementary tool, computational modeling constitutes the central framework of the present study.

Importantly, despite the rapid expansion of bioinformatic tools, relatively few studies have reported the experimental validation of aptamers designed primarily or exclusively through in silico pipelines, highlighting the need for integrative computational–experimental demonstrations.

In this work, we present the rational design, structural modeling, and experimental assessment of ssDNA aptamers targeting the SARS-CoV-2 S protein, using a computational-first strategy. Beyond its clinical relevance, SARS-CoV-2 provides a structurally well-characterized and extensively studied model system for testing digital aptamer engineering workflows. By combining protein and nucleic-acid structural data with computational methods, we designed two 20-nucleotide ssDNA aptamers, named seq_2714 and seq_1397, capable of recognizing the RBD domain of the S protein. This study illustrates how bioinformatics-driven approaches can streamline candidate prioritization, reduce experimental attrition, and establish a scalable platform adaptable to emerging infectious agents and other biomedical targets.

## 2. Materials and Methods

### 2.1. Bioinformatic Design of ssDNA Aptamers for the Recognition of the SARS-CoV-2 S Protein

The in silico methodology for designing ssDNA aptamers consist of two general parts: structural modeling of the ssDNA molecules and their docking to the receptor (Figure 1). For the first part, the methodology used consists of four steps, following the reported considerations [14,15]: generation of the sequence library (1), secondary structure prediction (2), tertiary structure prediction (3), and tertiary structure optimization (4) (Figure 1, step I).

First (1), a library of 4000 random ssDNA sequences, each 20 nucleotides long with a percentage of CG of 60%, was designed using in-house Python3 scripts (Python Software Foundation; https://www.python.org/). The initial pool size of 4000 sequences was selected as a computationally manageable starting space for this proof-of-principle workflow, allowing the complete screening procedure to be performed using standard desktop computing resources. This pool was not intended to reproduce the sequence diversity of conventional SELEX libraries but rather to evaluate the feasibility of successive computational filtering and prioritization of candidate aptamers. The initial sequence-generation parameters were selected as practical design constraints rather than on the basis of previously reported SARS-CoV-2-specific aptamers, which were not available when the computational workflow was initially developed. A length of 20 nucleotides was chosen to generate relatively short ssDNA sequences while retaining sufficient sequence complexity for secondary and tertiary structure formation and facilitating subsequent synthesis and experimental handling. This approach is consistent with general principles of aptamer optimization, in which sequences are frequently minimized while preserving their functional structure and target-recognition properties [16].

Then (2), the secondary structure of the aptamers was predicted using the free-energy minimization method implemented in FOLD [17], part of the RNAstructure program [18].

FOLD was also used to calculate the folding free energy at 37 °C for each secondary structure, selecting the 100 sequences with the lowest free energy. Since, to date, there is no tertiary structure prediction method for ssDNA [14], thymine bases were replaced with uracil in these 100 structures to use the RNA Composer web server [19,20] for tertiary structure prediction (3). Ten three-dimensional models were calculated for each sequence, resulting in a total of 1000 structures. Finally, for the structural optimization (4), uracil bases were replaced with thymine and ribose sugars with deoxyribose using the psfgen plugin of the VMD program version 1.9.4a38 [21] and the CHARMM force field [22]. Subsequently, they were subjected to 1000 minimization steps at 37 °C in a solvation box with a concentration of 1.5 mM MgCl_2_. Finally, the ten structures with the lowest potential energy after minimization were selected.

The second part of the protocol is based on research by Soon & Aina Nordin [23] (Figure 1, step II). In this section, molecular docking between aptamers and S proteins was performed using the AutodockVina program (v1.1.2) [24]. For this, the ten best-designed aptamer structures from the previous section were used, along with three structures of the S protein: in the closed state (CS) (PDB-ID: 7DWY), open state (OS) (PDB-ID: 7KMS), and, presumably, in a transition state (TS) (PDB-ID: 6XM0). These were prepared for molecular docking using the CHARMM-GUI server [25] and AutodockTools (v1.1.2) [26], while the aptamers were prepared solely with AutodockTools. The calculation box for each case included the residues relevant for the interaction of the S protein with the ACE2 [27,28]. In the case that the aptamers present several structures, prior to molecular docking, structural clustering was performed, classifying groups with structures having an RMSD (structural similarity measure) of less than 2Å. Finally, the structure with the lowest potential energy was chosen as the representative of the group for molecular docking. The docking model complexes were subjected to a visual inspection based on the information pertaining to the relevant residues of the S protein involved in ACE2 binding [29,30].

### 2.2. Expression and Purification of Recombinant SARS-CoV-2 RBD

For production and purification of the recombinant SARS-CoV-2 RBD protein, the recombinant SARS-CoV-2 RBD protein used in this study was produced in-house from a synthetic gene obtained from GenScript. The RBD sequence was based on the Wuhan-Hu-1 SARS-CoV-2 reference strain (GenBank accession number: NC_045512.2). The recombinant protein was expressed and purified according to the established protocol described below. Additionally, commercially purified recombinant RBD protein from Rekon Biotech was used for complementary biochemical validation experiments.

The gene encoding the RBD of the SARS-CoV-2 S protein was commercially synthesized and cloned into pET28a with a C-terminal His_6_-tag. *E. coli* BL21 (DE3)-pRIL cells were transformed with the plasmid construct and plated on LB agar supplemented with kanamycin (30 µg/mL) and chloramphenicol (34 µg/mL).

A single colony was grown overnight in LB containing antibiotics at 37 °C (180 rpm). The preculture was diluted into 200 mL LB medium with antibiotics and incubated at 30 °C (275 rpm) until the OD_600_ reached 0.3–0.4. Protein expression was induced with 0.5 mM IPTG and continued at 18 °C for 24 h (150 rpm). Cells were harvested by centrifugation (5000 rpm, 5 min, 4 °C) and stored at −20 °C.

All purification steps were performed at 4 °C. Pellets were resuspended in lysis buffer (20 mM Tris-HCl pH 7.9, 500 mM NaCl) supplemented with 200 µM PMSF and lysed by sonication (5 × 10 s pulses, 70% amplitude). After centrifugation (10,000 rpm, 30 min, 4 °C), the clarified lysate was incubated with Ni-NTA resin pre-equilibrated in washing buffer (20 mM Tris-HCl pH 7.9, 500 mM NaCl, 10 mM imidazole) for 1 h under gentle rotation.

The resin was washed with 20 column volumes of washing buffer, and bound protein was eluted using elution buffer (20 mM Tris-HCl pH 7.9, 500 mM NaCl) containing increasing imidazole concentrations (25–500 mM). Eluted fractions were analyzed by 10% sodium dodecyl sulfate–polyacrylamide gel electrophoresis (SDS-PAGE) and visualized with Coomassie Brilliant Blue.

Purified protein was dialyzed for 24 h at 4 °C against dialysis buffer (1× phosphate-buffered saline (PBS), 20% glycerol, 5% glucose, distilled water to volume), quantified by absorbance at 280 nm (NanoDrop™ One, Thermo Fisher Scientific, Wilmington, DE, USA), and stored at −80 °C.

### 2.3. Western Blot

Recombinant SARS-CoV-2 S protein (0.3 mg/mL) was resolved by SDS-PAGE under denaturing conditions using a 15% resolving acrylamide gel. Electrophoresis was conducted at a constant voltage of 120 V until adequate protein separation was achieved.

Following electrophoretic separation, proteins were equilibrated in 1X PBS and electrotransferred onto a membrane using the Trans-Blot^®^ Turbo™ Transfer System (Bio-Rad Laboratories, Hercules, CA, USA), according to the manufacturer’s instructions.

To prevent nonspecific binding, the membrane was blocked with 1% bovine serum albumin (BSA) in PBS for 30 min at room temperature under gentle agitation. After removal of the blocking solution, biotinylated aptamers (seq_1397 and seq_2714), previously denatured and allowed to refold, were incubated with the membrane at a final concentration of 100 nM overnight at 4 °C with gentle agitation to facilitate specific binding to the immobilized S protein.

Following incubation, the membrane was washed three times for 3 min each with PBS containing 0.05% Tween-20 (PBS-T) to remove the unbound aptamer. Detection was performed by incubation with streptavidin–horseradish peroxidase (HRP) conjugate diluted 1:10,000 for 45 min at room temperature. After additional washing steps with PBS-T, signal development was achieved using 3,3′-diaminobenzidine (DAB) as chromogenic substrate, allowing for visualization of the aptamer–S protein interactions.

### 2.4. Western Blot Analysis of seq_2714 Recognition of Recombinant RBD and Unrelated Proteins

To further evaluate the specificity of the selected aptamer, Western blot analysis was performed using biotinylated ssDNA aptamer seq_2714. Recombinant SARS-CoV-2 RBD containing a 6 × His tag was used as the target protein, while unrelated proteins, including His-tagged phi29 DNA polymerase from bacteriophage and His-tagged NS5 polymerase from West Nile virus, were included as protein controls. BSA and a complex protein extract from *Acanthamoeba* (EA) were included as additional unrelated protein and biological matrix controls, respectively.

A total amount of 9 µg of each protein sample was separated by SDS-PAGE under denaturing conditions and subsequently transferred onto a nitrocellulose membrane. Following transfer, membranes were blocked with 1% BSA in PBS to prevent nonspecific interactions. Membranes were incubated with biotinylated seq_2714 at a final concentration of 100 nM under gentle agitation at room temperature.

After incubation, membranes were washed with PBS-T to remove the unbound aptamers. Aptamer binding was detected using HRP conjugate, followed by signal development using an appropriate chromogenic or chemiluminescent detection system.

### 2.5. Dot Blot

To evaluate the analytical sensitivity of the selected aptamers, serial dilutions of recombinant SARS-CoV-2 S protein were prepared at the following concentrations: 1.3 mg/mL, 0.13 mg/mL, 0.026 mg/mL, 0.013 mg/mL, 1.3 × 10^−3^ mg/mL, and 1.3 × 10^−4^ mg/mL.

Aliquots of each dilution were directly applied onto a nitrocellulose membrane using a vacuum-assisted dot blot apparatus to ensure uniform protein immobilization. After fixation, the membrane was air-dried and blocked with 1% BSA in PBS to prevent nonspecific binding.

Three parallel conditions were evaluated to determine the detection limit of the aptamers: negative control—membrane processed without incubation with aptamers; aptamer seq_2714—biotinylated aptamer incubated at 1 µM; and aptamer seq_1397—biotinylated aptamer incubated at 1 µM.

Following incubation under gentle agitation at room temperature, membranes were washed with PBS-T 0.05% to remove the unbound aptamers.

Detection was carried out by incubation with streptavidin–HRP conjugate diluted 1:10,000. After washing steps with PBS-T, signal development was performed using DAB as a chromogenic substrate, allowing for visualization of the aptamer–S protein interactions.

The intensities of the dots obtained in the dot blot assays were quantified using ImageJ 2 software (National Institutes of Health, Bethesda, MD, USA). Signal intensities were measured by densitometric analysis and normalized against background values to allow for semi-quantitative comparison among samples.

### 2.6. Evaluation of Aptamer Specificity by Dot Blot Analysis

To further characterize the recognition profile and specificity of the selected aptamer, an additional dot blot assay was performed using biotinylated ssDNA aptamers seq_2714 and seq_1397. Two recombinant SARS-CoV-2 RBD preparations were evaluated: an in-house produced recombinant RBD [RBD(1)], expressed and purified from a synthetic RBD gene obtained from GenScript, and a commercial recombinant SARS-CoV-2 Spike S1 receptor-binding domain (RBD) protein (Rekom Biotech S.L., Spain; catalogue no. RAG0074), expressed in Pichia pastoris, was also used. The manufacturer confirmed that the recombinant protein contains a His-tag; however, the exact construct boundaries and tag configuration were not disclosed by the manufacturer for confidentiality reasons.

To assess target specificity, an anti-RBD antibody was included as an independent positive control for RBD detection. His-tagged unrelated recombinant proteins, including phi29 DNA polymerase from bacteriophage and NS5 polymerase from West Nile virus, were used as protein controls. In addition, bovine serum albumin (BSA) and a complex protein extract from *Acanthamoeba* were included as unrelated protein and biological matrix controls, respectively.

Protein samples were immobilized onto nitrocellulose membranes at a concentration of 0.013 mg/mL using a vacuum-assisted dot blot apparatus. After protein immobilization and membrane blocking with 1% BSA in PBS, membranes were incubated with biotinylated seq_2714 (1 µM) and seq_1397 (1 µM) under gentle agitation at room temperature. Following incubation, membranes were washed with PBS containing 0.05% Tween-20 (PBS-T) to remove the unbound aptamers. Aptamer binding was detected using streptavidin–horseradish peroxidase (HRP) conjugate (1:10,000 dilution) followed by signal development using DAB as a chromogenic substrate.

For independent RBD detection, membranes were incubated with anti-RBD antibody (0.2 mg/mL) (SARS-CoV-2, spike antibody(1A9), GENTEX) followed by the corresponding antibody detection system according to the manufacturer’s recommendations.

#### 2.6.1. Clinical Samples

Saliva samples were collected from patients who tested positive for the SARS-CoV-2 antigen on the day of the sample collection. Immediately after collection, samples were treated with freshly prepared dithiothreitol (DTT; Sigma-Aldrich, catalog no. 20-265) to reduce mucus viscosity and facilitate homogenization prior to analysis.

The study protocol involving the use of human biological samples was reviewed and approved by the corresponding Institutional Ethics Committee (approval code: 20.10.1692-GHM). All procedures were conducted in accordance with the Declaration of Helsinki and applicable national regulations governing research involving human subjects.

#### 2.6.2. Environmental Samples

A total of two drinking water treatment plants (DWTPs) and three wastewater treatment plants (WWTPs) were selected. Samples were collected in a maximum of 100 L in the case of the DWTPs, and in the case of the WWTPs the maximum volume was 50 L. In all cases, the water samples were concentrated using the IDEXX^®^ Filta-Max system (IDEXX Laboratories, Westbrook, ME, USA) according to the manufacturer’s instructions and following the US Environmental Protection Agency (US EPA) method 1623 [31] for the detection of *Acanthamoeba* and the CSIC protocol for the concentration of free SARS-CoV-2 in water. Finally, a total of 5 mL of each concentrated sample was eluted and fractionated for different analyses.

Conventional PCR for *Acanthamoeba* detection (KAPA Taq HotStart PCR Kit-KK1511, KAPA BIOSYSTEMS^®^, Wilmington, MA, USA) was performed according to the cycling and conditions described by the manufacturer. PCR reactions were set up for a final volume of 25 µL, using 5 µL of DNA, 1.5 mM MgCl_2_, 1× of KAPA Taq HotStart buffer, 0.2 mM dNTP Mix, 0.5U KAPA Taq HotStart DNA polymerase, 0.20 µM of each primer (JDP1-forward: 5′-GGCCCAGATCGTTTACCGTGAA; JDP2-reverse: 5′-TCTCACAAGCTGCTAGGGAGTCA) and MQ-UV water to make up the volume. Amplification cycles were performed on an Mx3000P thermal cycler (Agilent Technologies, Santa Clara, CA, USA) according to the following protocol: 1 min pre-denaturation at 95 °C, followed by 45 cycles with 30 s denaturation at 95 °C, 30 s hybridization at 60 °C and 1 min elongation at 72 °C. Finally, a 10 min elongation at 72 °C. DNA-free water was used as a negative control and DNA extracted from amoebae from the culture unit of the San Pablo CEU University of Madrid was used as a positive control. It was resolved by electrophoresis in 1.5% agarose gel [32].

For the detection of free virus, RTq-PCR (One Step PrimeScript TM RT-PCR Kit, TAKARA^®^ Bio USA, Mountain View, CA, USA) was performed according to the protocol of the VATar COVID-19 project of the CSIC. It was performed against three different viral targets (E gene, IP4 and N1/N2) according to the cycling and conditions described by the manufacturer. Reactions were set up for a final volume of 10 µL, using 2.5 µL of DNA, 1× One Step RT-PCR buffer, 0.5U Takara Ex Taq HotStart polymerase, PrimeScript RT enzyme Mix, 0.2 µM of each primer and probe, 50X ROX and RNAase-free water to make up the volume. Amplification cycles were performed on the QuantStudio 3 real-time PCR thermal cycler (Applied Biosystems^®^, Thermo Fisher Scientific, Waltham, MA, USA) according to the following protocol for viral targets E and IP4: 20 min retrotranscription at 55 °C and 3 min denaturation at 95 °C, followed by 50 PCR cycles of 15 s at 95 °C and 30 s at 58 °C; for the N target: 15 min retrotranscription at 50 °C and 2 min denaturation at 95 °C, followed by 45 PCR cycles of 3 s at 95 °C and 30 s at 55 °C. RNA-free water was used as a negative control, and RNA extracted from a plasmid expressing for protein E, IP4 and N was used as a positive control. Samples were considered positive when a Ct of less than 40 was obtained in at least two of the three viral targets, according to the manufacturer’s indications.

## 3. Results

### 3.1. Bioinformatic Design of Aptamers for the Recognition of the S Protein

**Figure 2:**
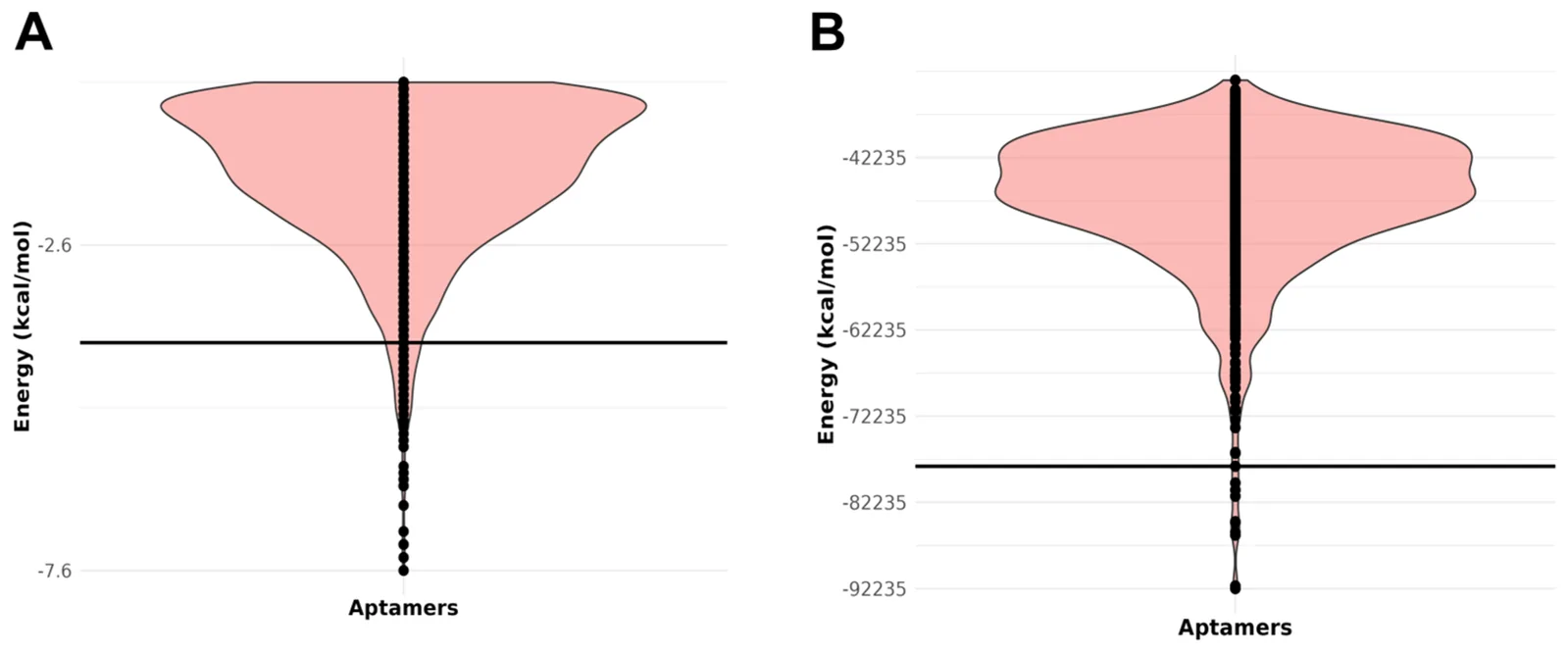
Energy violin plot of the secondary and tridimensional structures of aptamers. (**A**) The distribution of energies associated with the secondary structures of the 4000 generated aptamers from our in-house library is shown. The horizontal bold black line stands for the −4.1 cutoff value. (**B**) The distribution of the potential energies associated with the 1000 modeled tertiary structures is showcased. The horizontal bold black line stands for the −78,051.7 cutoff value.

Regarding the generation of the aptamer library, 4000 non-repetitive and random ssDNA sequences of a length of 20 nucleotides and a CG percentage of 60% were successfully produced. The percentages of adenine and thymine varied between 0% and 40%. Additionally, the 4000 secondary structures with their associated free energy values were generated (Figure 2A). Consequently, the sequences were ordered by free energy value, with the highest being −0.1 kcal/mol and the lowest −7.6 kcal/mol. However, the 100 sequences with the most favorable energies were selected, using a cutoff energy value of −4.1 kcal/mol. Ten three-dimensional models were obtained for each sequence, yielding a total of 1000 structures, which were subsequently optimized. The potential energy values associated with these structures range from −92,234.9 kcal/mol to −33,273.5 kcal/mol. From these optimized structures, 100 aptamer models with the lowest potential energy were selected using a cutoff of −78,051.7 kcal/mol (Figure 2B).

**Figure 3:**
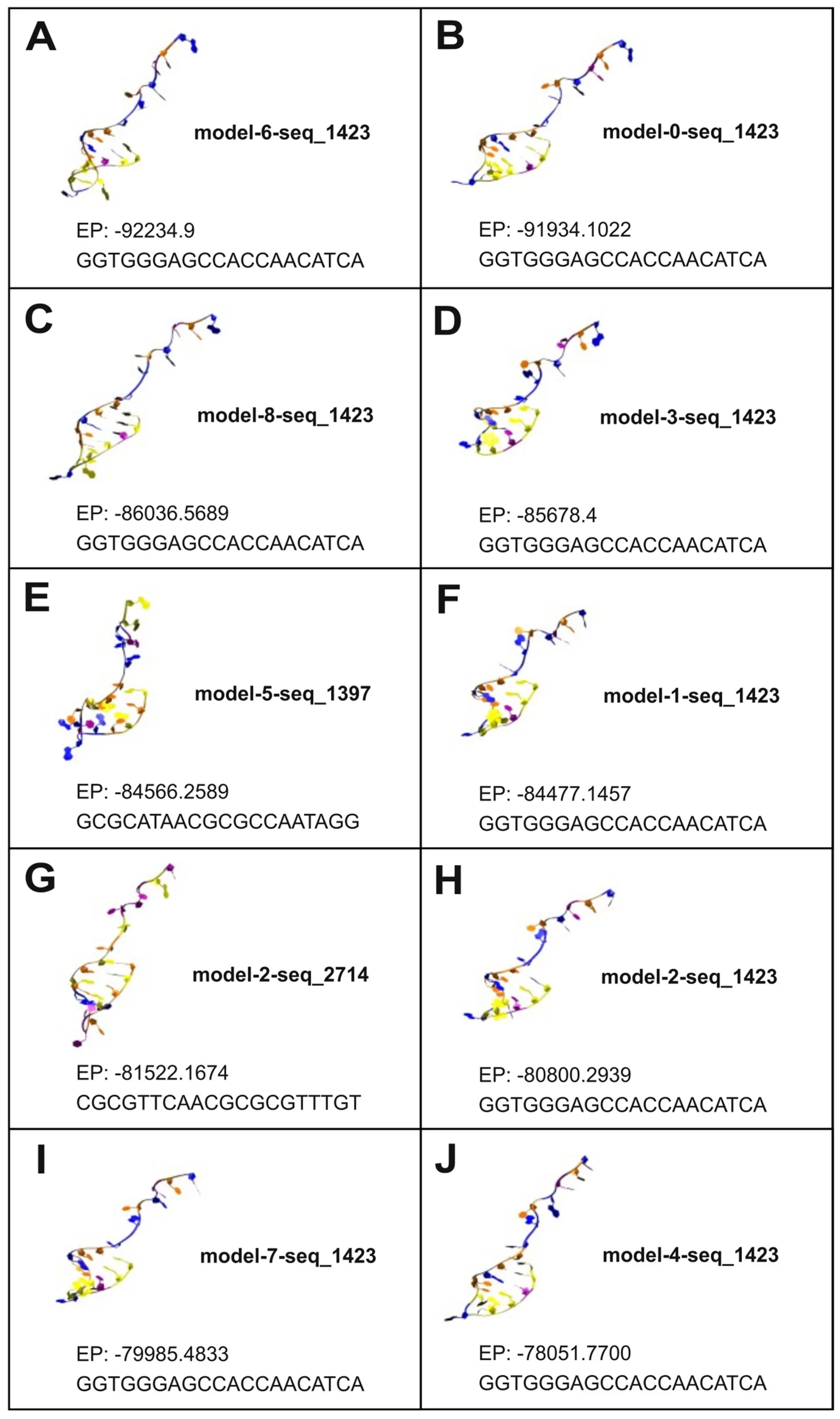
The ten most stable aptamer structures. The tertiary structures of the aptamers that presented the lowest potential energy are shown in ribbon style. The name of each aptamer code is (seq_[sequence number]), and the suffix modelis used to differentiate the various models generated in the tertiary structure. The DNA sequences of each aptamer are detailed. The nucleotides’ adenine, cytosine, guanine and thymine are colored in blue, orange, yellow and violet, respectively. EP: potential energy, in kcal/mol.

Subsequently, they were ordered based on their potential energy, and the top ten are shown in Figure 3. It is important to note that sequence number 1423 appears eight times in the group but with different structures, which may suggest that it is stable in various conformations.

**Figure 4:**
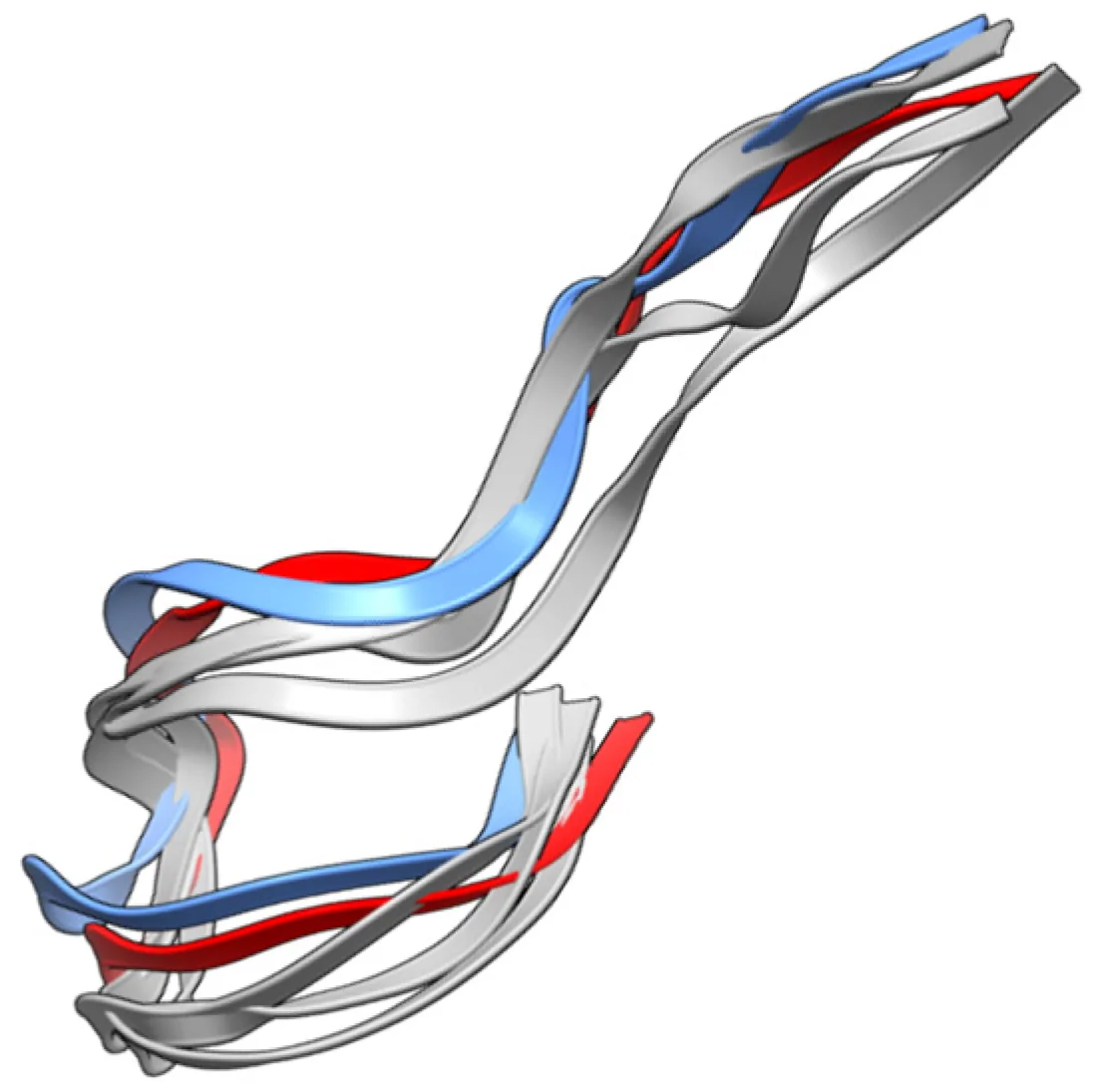
Structural clustering of models for seq_1423. The structural clustering of the models obtained for sequence number 1423 is shown as an illustration. The structures colored in blue and red correspond to two groups. However, the structures in gray do not belong to any group, and, therefore, each of them is considered distinct from the others for docking purposes. The structural superposition was performed using VMD software, version 1.9.4a38.

Sequence number 1423 presents several structural models; therefore, clustering of similar ones was performed. Figure 4 shows the groups, where model-6-seq_1423 was the representative of the blue group, model-2-seq_1423 of the red group, and the structures model-8-seq_1423, model-3-seq_1423, and model-1-seq_1423 were treated as distinct, as they were not grouped into any cluster. Thus, seven structures were selected for molecular docking (see Table 1).

Each structure was docked to the SARS-CoV-2 S protein. This viral protein plays a key role in receptor recognition and membrane fusion and is composed of two subunits: S1 (residues 14–685), which mediates the former, and S2 (residues 686–1273), which mediates the latter function. Here, we focused on the S1 subunit, which contains the RBD (residues 319–541). This domain binds to the host receptor ACE2, initiating structural rearrangements of the S protein that allow the virus to fuse with the host cell membrane. Cryo-electron microscopy (cryo-EM) structures of the S protein at atomic resolution reveal that the RBD adopts open and closed conformations, and that the S protein binds to the ACE2 when the RBD is in the open state [33,34].

Although the ACE2 interacts with the RBD in open state, molecules recognizing the closed RBD are also relevant, as stabilization of this conformation could alter the transition to the open state required for viral entry. In addition, targeting intermediate or transitional RBD conformations between the closed and open states may interfere with the conformational dynamics of the S protein required for viral fusion, thereby contributing to inhibition of viral infectivity. Thus, we performed docking of the seven structures listed in Table 1 in the RBD OS, CS and a TS. The template tertiary structures of the target correspond to the following cryo-EM structures for the open, closed and transitional states: PDB IDs 7KMS, 7DWY, 6XM0, respectively. Table 1 summarizes the docking scores for each aptamer across the different RBD conformational states and their average values. The seq_2714 structure showed the most negative docking scores for the open (OS, −9.5 kcal/mol) and transitional (TS, −10.1 kcal/mol) states and ranked third for the closed state (CS, −8.7 kcal/mol). Overall, seq_2714 exhibited the most favorable average docking score across the three independently evaluated S protein conformational models and was, therefore, prioritized for subsequent experimental testing. Seq_1423 ranked second, whereas seq_1397 showed comparatively less favorable average docking scores.

**Figure 5:**
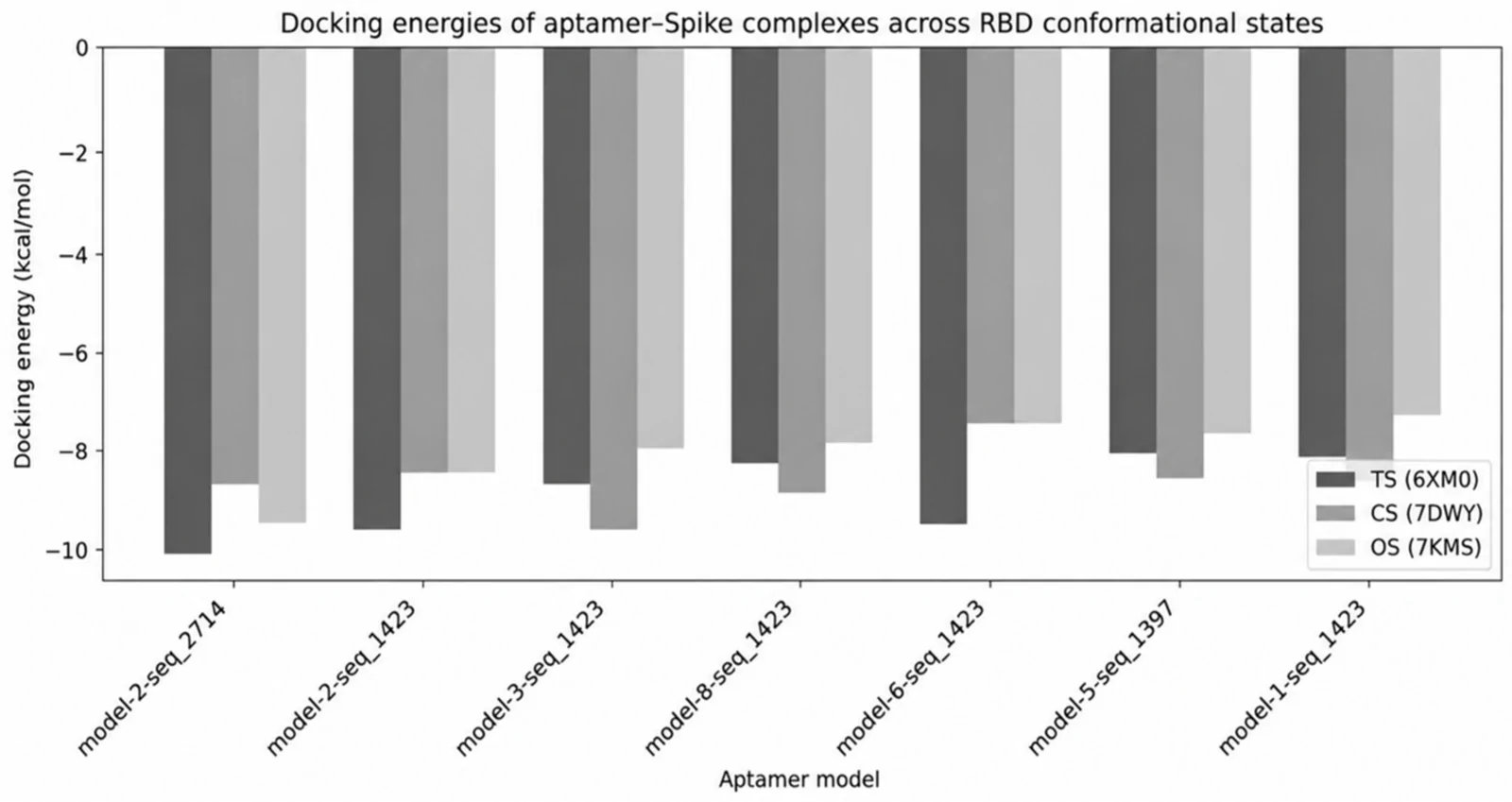
Docking scores of prioritized ssDNA aptamer–S protein complexes across different RBD conformational states.

To facilitate comparison of aptamer performance across the different RBD conformational states, docking scores are additionally represented graphically in Figure 5. This visualization shows the relative binding profiles of the seven prioritized aptamer models across the three evaluated RBD states (TS, CS, and OS). Among the candidates, seq_2714 displayed the most favorable average predicted docking score, with consistently strong interactions across the different conformational states.

**Figure 6:**
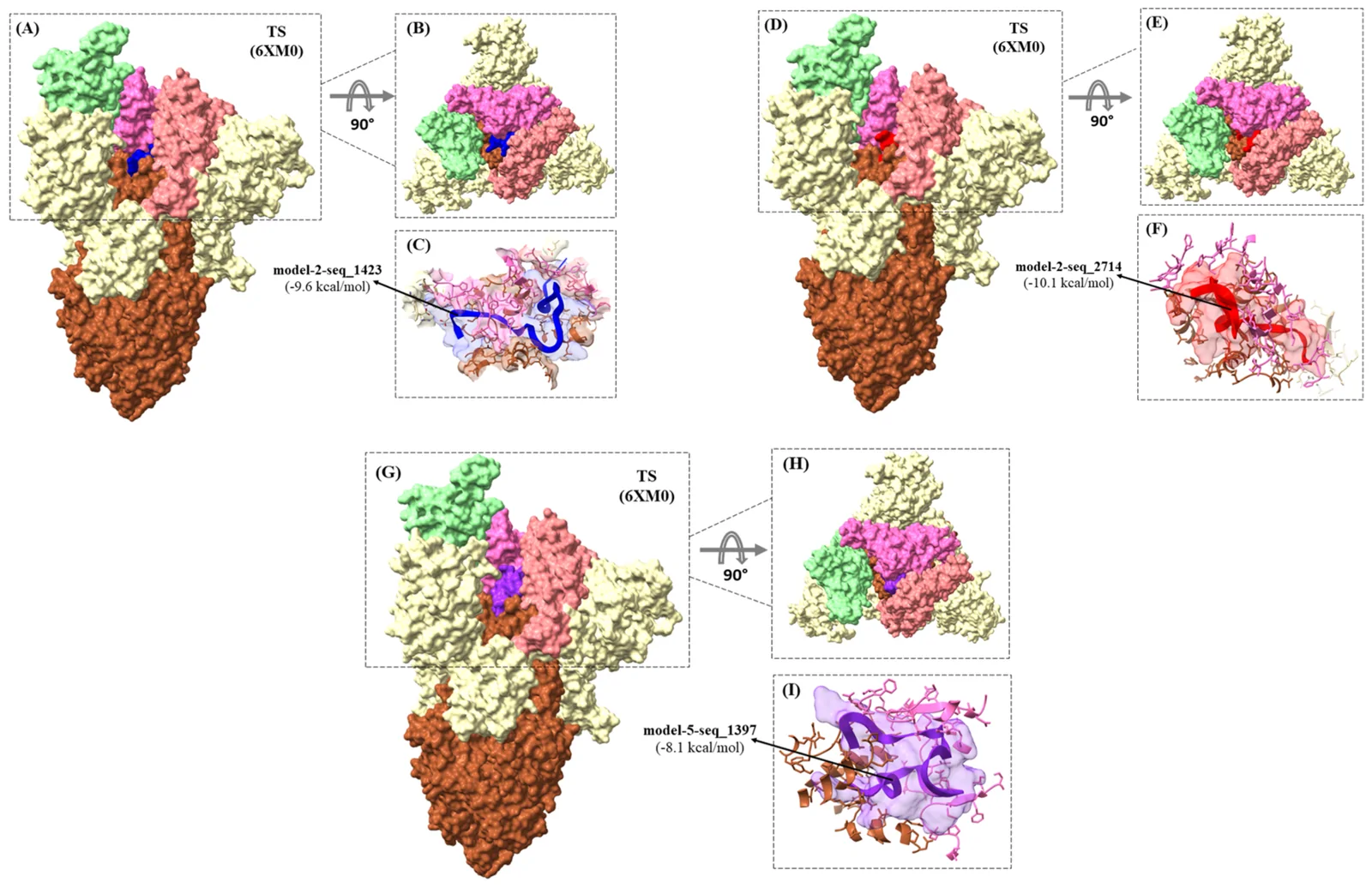
Aptamer–S protein TS complex. The trimeric S protein in its transitional state (TS) is shown in surface representation. The three RBDs of each chain are colored in pink, green and salmon, while all NTD domains are shown in light yellow and all S2 subunits are highlighted in brown. The three aptamers are shown as surface: seq_2714 (red), seq_1423 (blue), and seq_1397-F and -I) the S protein residues within 5 Å of aptamers are shown as sticks, while the corresponding aptamers are shown in cartoon and transparent surface. The docking score for each aptamer is also indicated as referred in Table 1.

**Figure 7:**
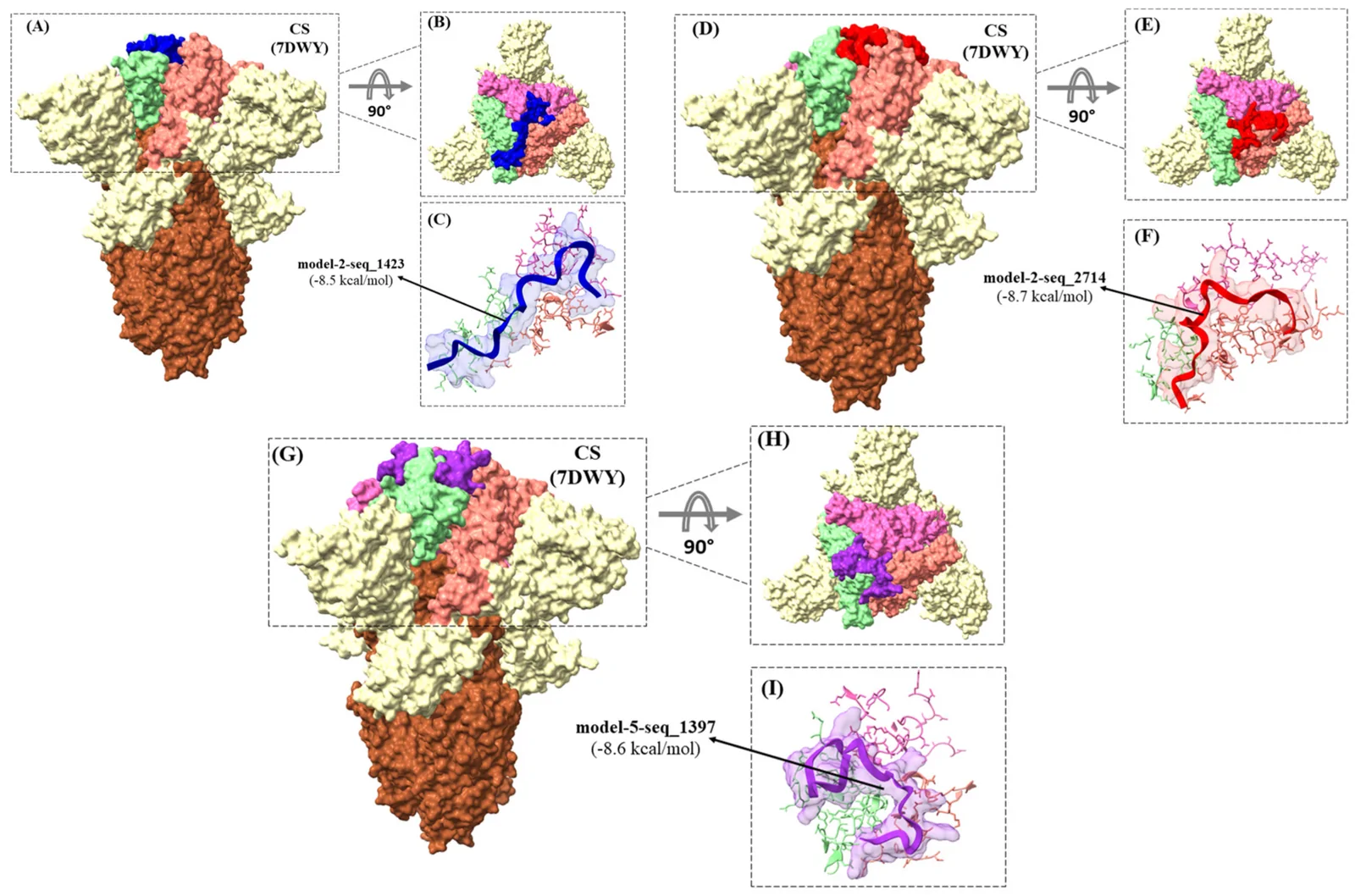
Aptamer–S protein CS complex. The trimeric S protein in its closed state (CS) is shown in surface. The three RBDs of each chain are colored in pink, green and salmon while all NTD domains are shown in light yellow and all subunit 2 are highlighted in brown. The three aptamers—seq_2714 (red), seq_1423 (blue), and seq_1397-F and -I—are shown as surface representations, and the S protein residues within 5 Å of the aptamers are shown as sticks, while the corresponding aptamers are shown as illustrations and transparent surface representations. The docking score for each aptamer is also indicated, as presented in Table 1.

**Figure 8:**
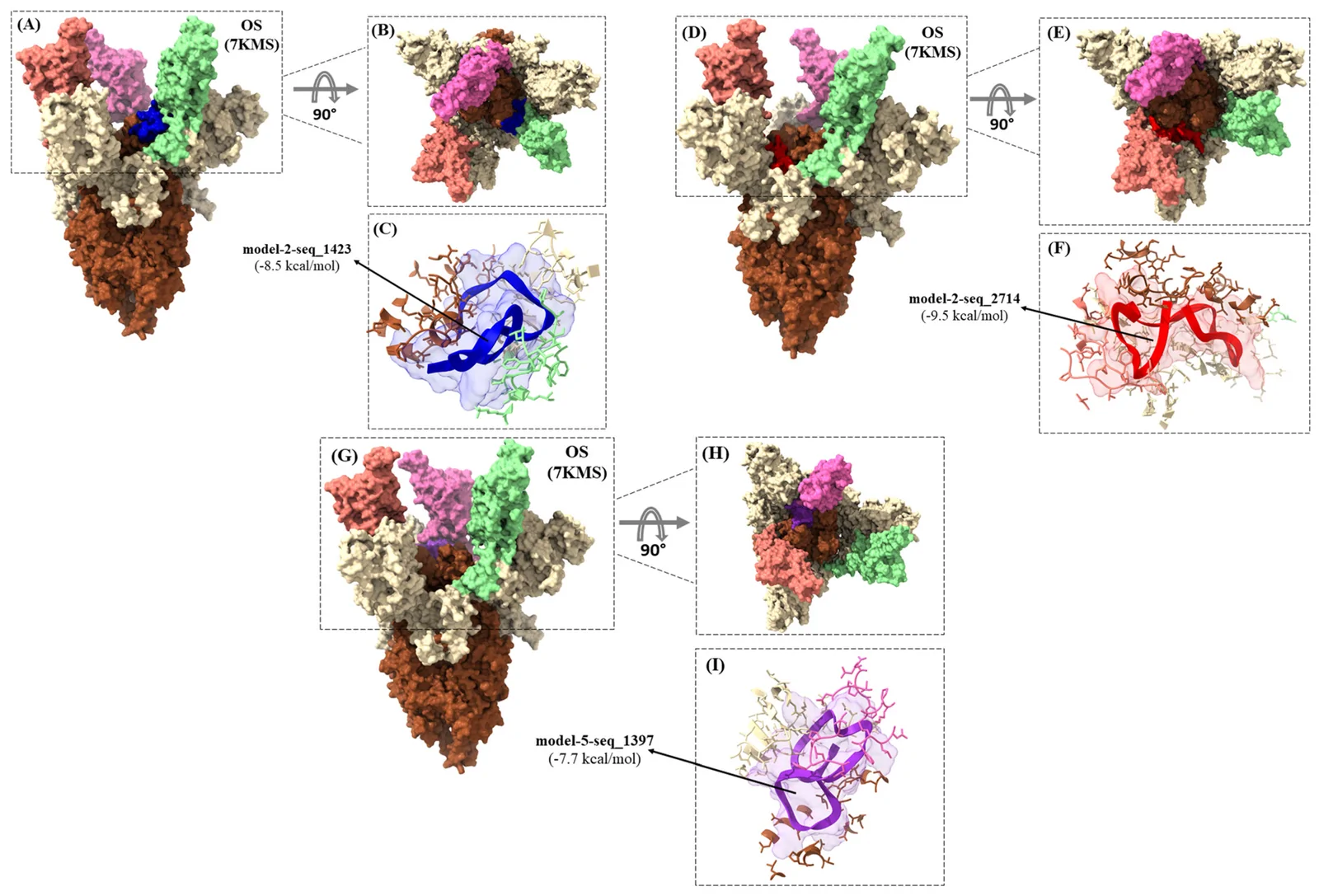
Aptamer–S protein OS complex. The trimeric S protein in its open state (OS) is shown as a surface representation. The three RBDs of each chain are colored in pink, green and salmon, while all NTD domains are shown in light yellow and all S2 subunits are highlighted in brown. The three aptamers—seq_2714 (red), seq_1423 (blue), and seq_1397-F and -I—are shown as surface representations, and the S protein residues within 5 Å of the aptamers are shown as sticks, while the corresponding aptamers are shown as illustrations and transparent surface representations. The docking score for each aptamer is also indicated, as presented in Table 1.

**Figure 9:**
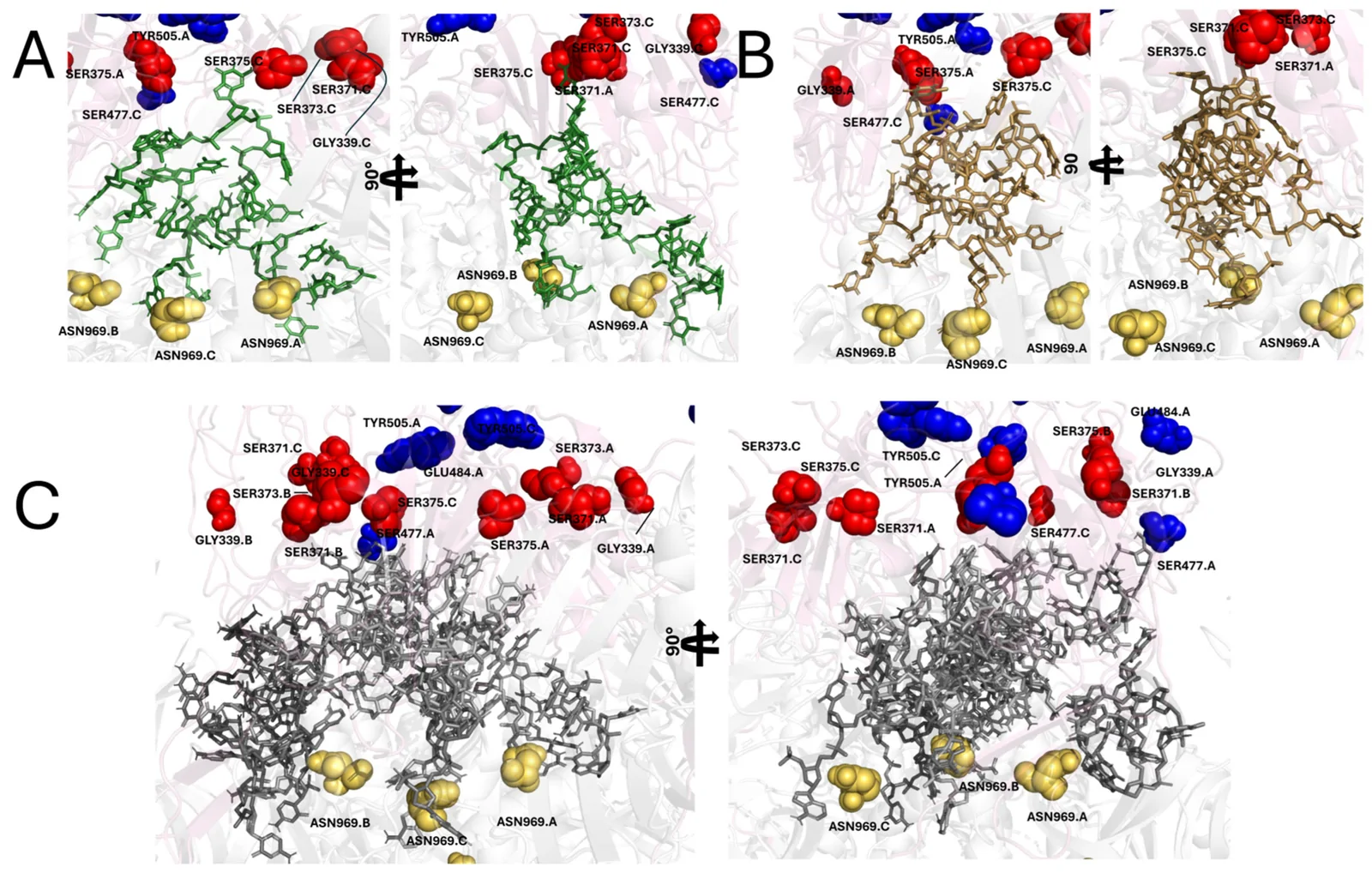
Aptamers’ binding poses in the S protein RBD transient-state conformation. The structural arrangements of aptamers ((**A**), forest green) seq_2714 and ((**B**), sand) seq_1397 and ((**C**), black), the different structures of seq_1423 are displayed as sticks. Residues belonging to the RBD, RBM and S2 subunit are shown in red, blue and orange–yellow spheres, respectively. Residues from the S protein are identified by their corresponding sequence numbering and respective chain.

The aptamer–S protein complexes for seq_2714, seq_1423 and seq_1397 targeting RBD OS, CS and TS are shown in Figure 6, Figure 7 and Figure 8. For docking, since the S protein is a trimer, we considered the three RBDs as potential targets for aptamer binding. Note that in the open state, structurally speaking, the RBD adopts an up conformation (Figure 9), whereas in the closed state the RBD remains buried in a down conformation (Figure 8).

Our docking predictions showed that aptamer poses vary depending on the RBD state (Figure 6, Figure 7 and Figure 8). When RBD adopts a TS, aptamers interact not only with the RBD but also with the S2 subunit (Figure 6). Meanwhile, in a CS, aptamers are located on the surface of the RBD (Figure 8). In the OS, aptamers are placed in an area flanked by the RBD and the S1A and S2 domains (Figure 9).

**Figure 10:**
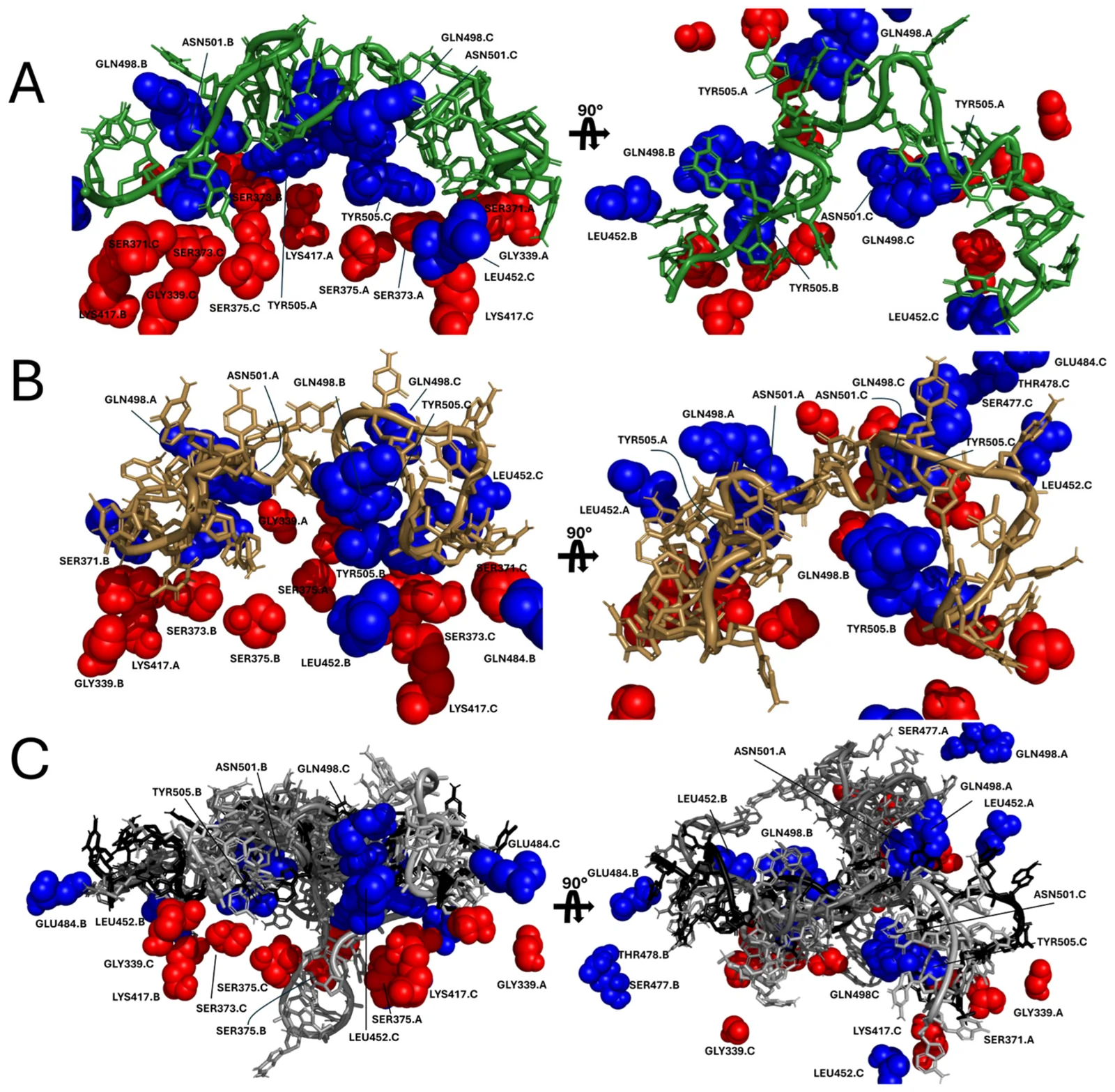
Aptamers’ binding poses in the S protein RBD closed-state conformation. Structural arrangements of aptamers ((**A**), forest green) seq_2714 and ((**B**), sand) seq_1397, as well as illustrating the alternative conformations of ((**C**), black) seq_1423, all rendered in stick representation. Residues from the RBD and RBM domains are represented as red and blue spheres, respectively. Residues from the S protein are identified by their corresponding sequence numbering and respective chain.

Also, molecular docking complexes were visually inspected to evaluate the proximity of the aptamers to the S protein residues relevant for infectivity (Figure 9 and Figure 10). Note that the RBD contains a subdomain critical for facilitating the binding of SARS-CoV-2 to the human ACE2 receptor, which is known as the receptor-binding motif (RBM). The RBM contains a total of 72 amino acids and is connected to the ACE2 receptor, particularly between residues 437 and 508 [35]. Within the RBM, six key amino acid residues—Leu-455, Phe-486, Gln-493, Ser-494, Asn-501, and Tyr-505—are responsible for enabling the virus to bind to ACE2 [28].

For the RBD in the TS, all aptamer structures are in proximity to RBD residues, including Ser-375 and Ser-371 (Figure 9A–C). In addition, residue Ser-373 is also in close proximity to model-5-seq_1397 and seq_1423 (Figure 9B,C), whereas Gly-339 is close to seq_1423 and seq_2714 (Figure 9A,C). Importantly, all aptamers are located near residues Ser-477 and Tyr-505 from the RBM motif (Figure 9A–C). In the cases of seq_1397 and seq_1423, residues Ser-375 and Glu-484 are also additionally in close proximity, respectively (Figure 9B,C). As previously referred to in Figure 6, Figure 7 and Figure 8, all aptamers are located near the S2 subunit, particularly near residue Asn-969 (Figure 9A–C).

Compared to the TS, the CS favors aptamer binding exclusively to the RBD and RBM and involves a larger number of residues from these domains (Figure 10A–C). In particular, aptamers show proximity to the same residues mentioned for the TS, with additional contacts involving Lys-417 from the RBD and residues Gln-498, Lys-452 and Asn-501 from the RBM (Figure 10A–C).

**Figure 11:**
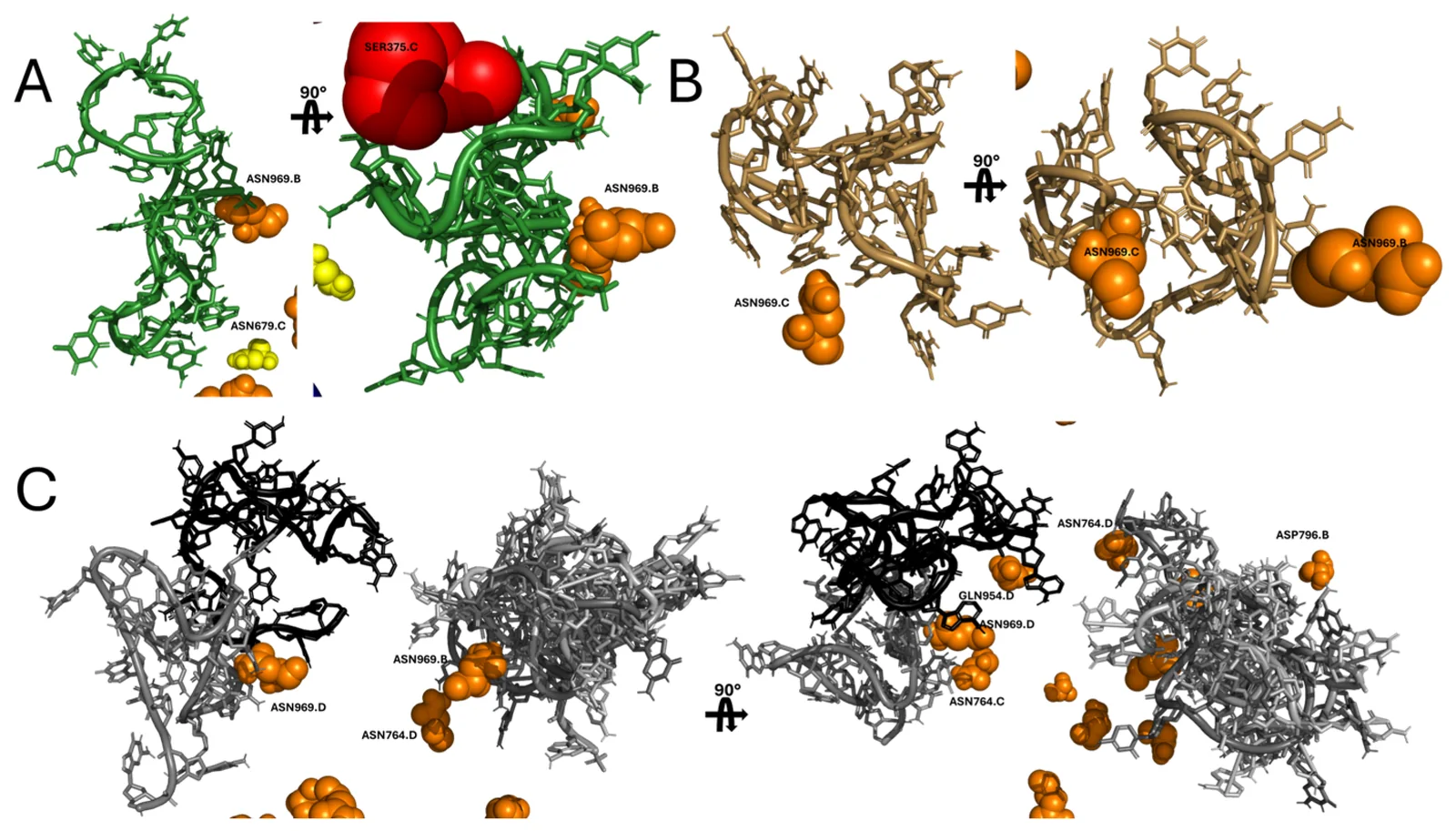
Binding poses of aptamers in the **S protein** RBD open-state conformation: ((**A**), forest green) seq_2714, ((**B**), sand) seq_1397, and ((**C**), black) alternative conformations of seq_1423, all depicted in stick representation. Residues from the S1/S2 interface and S2 domains are represented as yellow and orange–yellow spheres, respectively.

Lastly, the RBD in its OS conformation evidence that aptamers are close to residues Asn-969 of the S2 domain (Figure 11A–C), and, in addition, the seq_1423 conformations are also near to residues Asn-764, Gln-954 and Asp-796 of the S2 domain (Figure 11C). Only seq_2714 is in proximity to the residue Ser-375 of the RBD domain (Figure 11A).

Based on the molecular docking results, the aptamer seq_2714 was selected for experimental validation, as it exhibited the most favorable average docking score (−9.4 kcal/mol) across the three evaluated S protein conformational states. In addition, seq_2714 adopts poses close to the RBD residues in all of the RBD conformational states compared with seq_1397 and seq_1423. This sequence was, therefore, considered the most promising candidate for specific recognition of the target protein.

In addition to this top-ranked candidate, seq_1397 (mean docking score = −8.1 kcal/mol) was selected as a second candidate with a comparatively less favorable docking score but a predicted binding mode similar to that of seq_2714.

This aptamer presented a different nucleotide composition and structural topology, which could influence its interaction mode with the S protein. Evaluating both aptamers, which showed different docking-score profiles, allowed for the assessment of whether variations in predicted binding strength would correspond to differences in recognition sensitivity or specificity during biochemical assays such as Western blot and dot blot analyses. Additionally, it is important to test an aptamer that interacts with a minimum number of residues that varies between strands, because it would be a stronger candidate to become a probe to detect several SARS-CoV-2 mutants.

### 3.2. Experimental Validation of Aptamer–S Protein Binding

**Figure 12:**
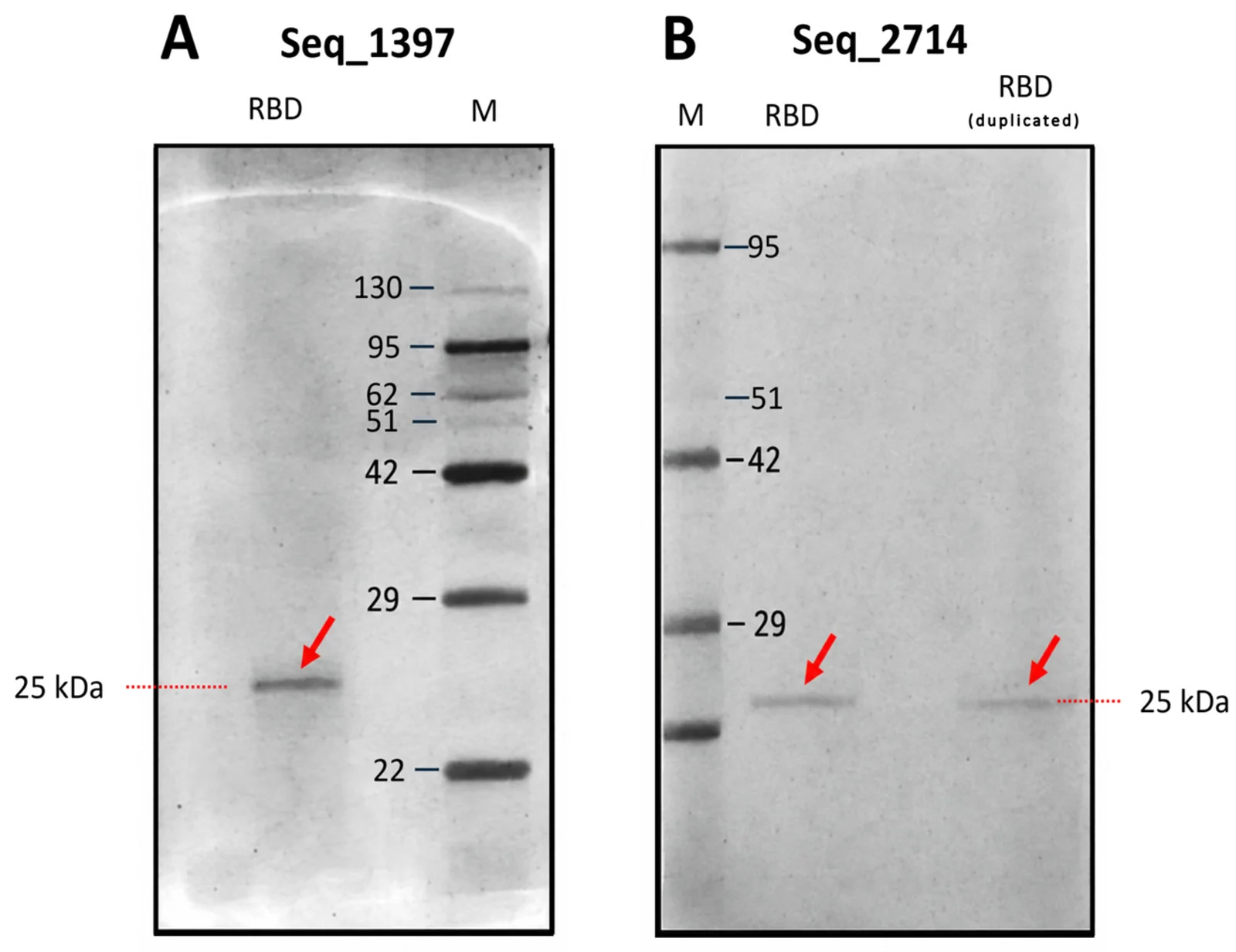
Western blot detection of recombinant SARS-CoV-2 S protein RBD using a biotinylated DNA aptamer. Purified recombinant S protein RBD (9 ug) was separated by SDS-PAGE (on a 12% polyacrylamide gel) under denaturing conditions and transferred onto a membrane. The membrane was incubated with biotinylated aptamers (**A**) seq_1397 (100 nM) and (**B**) seq_2714(100 nM), followed by detection with streptavidin–HRP and chromogenic development using DAB. The aptamer detected a band with an apparent molecular weight between 22 and 29 kDa, consistent with the expected molecular weight of the recombinant SARS-CoV-2 S protein RBD used in this study (theoretical molecular weight: 31.39 kDa).

The binding capacity of the prioritized aptamers toward the SARS-CoV-2 S protein RBD was experimentally evaluated using Western blot analysis. In the initial experimental assessment, seq_1397 was selected as a representative candidate among the prioritized aptamers and showed recognition of the recombinant SARS-CoV-2 RBD. Additionally, the top-ranked candidate seq_2714, selected based on its most favorable predicted docking profile, was also evaluated, and its ability to recognize the target protein under the same experimental conditions was confirmed (Figure 12).

For the Western blot analysis, a total of 9 µg of purified recombinant S protein RBD protein was resolved by SDS-PAGE on a 12% polyacrylamide gel and subsequently transferred onto a membrane. The membrane was incubated with the biotinylated aptamers seq_1397 or seq_2714 (100 nM), followed by detection using streptavidin–HRP and chromogenic development. A distinct band corresponding to the expected molecular weight of the recombinant RBD protein (~25 kDa) was detected. These results demonstrate that both aptamers retain their ability to recognize the recombinant RBD under denaturing electrophoretic conditions. However, since the Western blot analysis does not preserve the native three-dimensional structure of the protein, these findings do not allow for conclusions regarding recognition of the conformational epitopes in the native RBD. Further studies under native conditions will be required to fully characterize the molecular basis of aptamer–RBD recognition.

To further evaluate the specificity of seq_2714 recognition, additional Western blot control experiments were performed using unrelated proteins. A commercially available recombinant SARS-CoV-2 RBD protein containing a 6 × His tag was used as the positive control. Two unrelated His-tagged recombinant polymerases were included as protein controls: phi29 DNA polymerase from a bacteriophage and NS5 polymerase from West Nile virus. In addition, bovine serum albumin (BSA) and a complex protein extract from *Acanthamoeba* were evaluated as unrelated protein and complex biological matrix controls, respectively.

**Figure 13:**
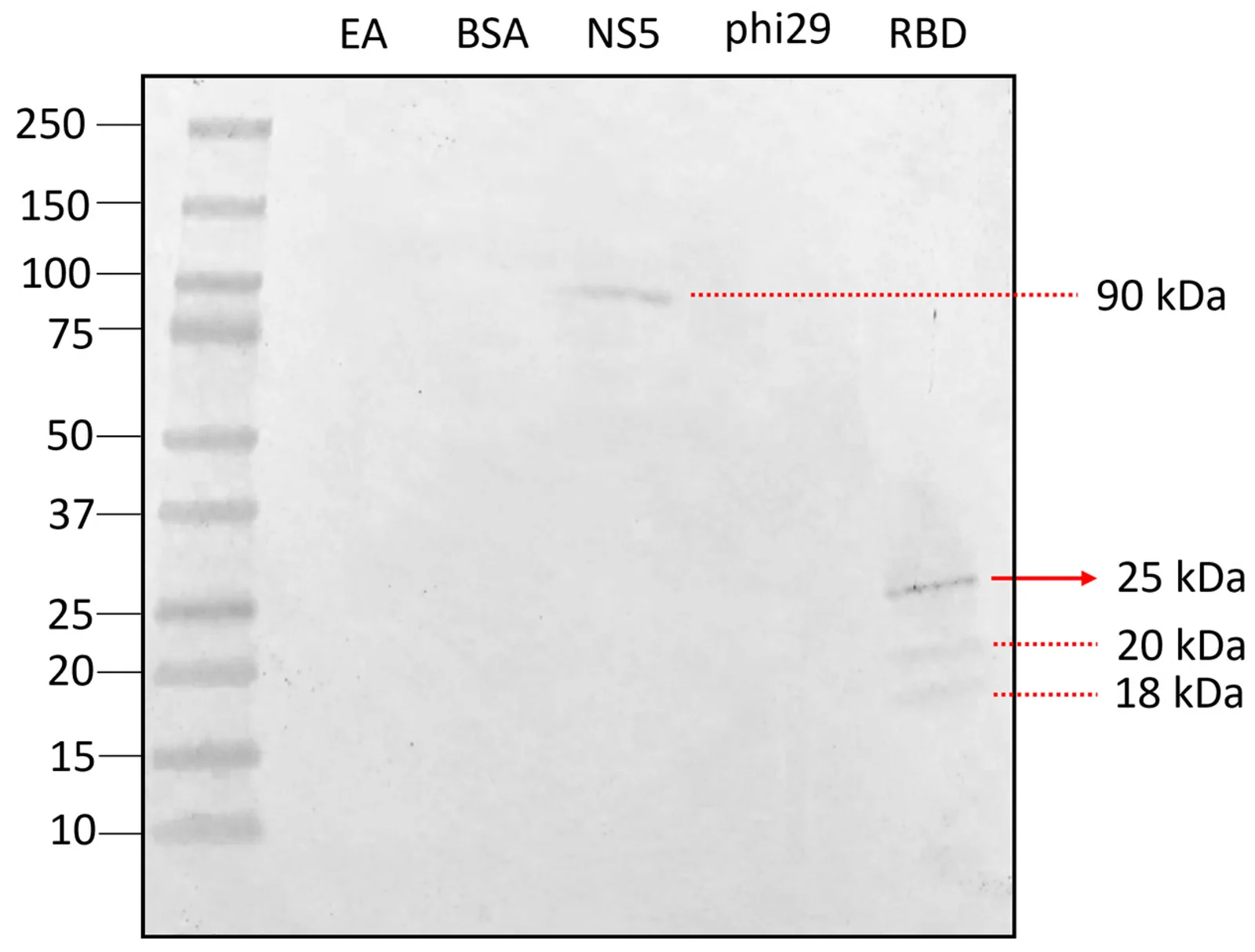
Western blot analysis of seq_2714 recognition of recombinant SARS-CoV-2 RBD and control proteins.

For these experiments, 9 µg of each protein sample were immobilized onto the membrane and incubated with the biotinylated aptamer seq_2714 (100 nM), followed by detection using the streptavidin–HRP-based system. As shown in Figure 13, seq_2714 produced a clear signal with the recombinant SARS-CoV-2 RBD. No detectable signal was observed with BSA, phi29 polymerase, or the *Acanthamoeba* protein extract. A weak signal was detected with the His-tagged NS5 polymerase control. These results indicate that seq_2714 preferentially recognizes recombinant SARS-CoV-2 RBD under the experimental conditions evaluated, although a minor interaction with unrelated proteins cannot be completely excluded.

Recombinant 6 × His-tagged SARS-CoV-2 RBD was used as a positive control, while phi29 DNA polymerase, West Nile virus NS5 polymerase, BSA, and *Acanthamoeba* extract (EA) were included as unrelated protein controls. A total of 9 µg of each sample was immobilized onto the membrane and incubated with biotinylated seq_2714 (100 nM). Detection was performed using streptavidin–HRP. Seq_2714 showed recognition of recombinant RBD, with only a weak signal observed for the NS5 polymerase control.

**Figure 14:**
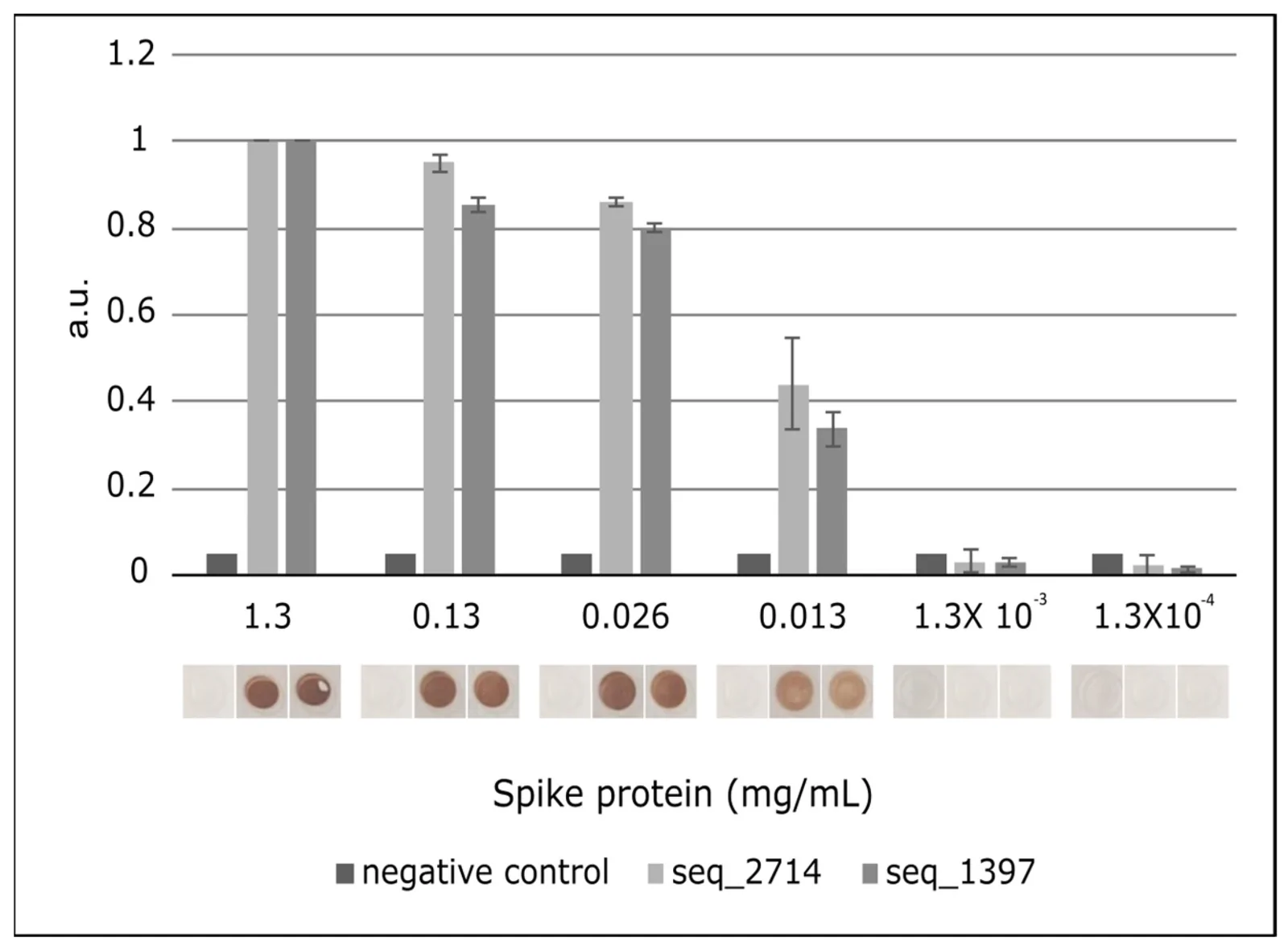
Dot blot analysis showing concentration-dependent recognition of the recombinant SARS-CoV-2 S protein by the selected aptamers. Serial dilutions of the recombinant S protein were immobilized on a nitrocellulose membrane and probed with the biotinylated aptamers seq_2714 and seq_1397 (1 µM). Detection was performed using streptavidin–HRP and chromogenic development with DAB. Both aptamers were able to detect the S protein across the tested concentration range, with a detectable signal observed down to approximately 0.013 mg/mL. Data are presented as the standard deviation (SD) of three independent experiments, each performed in triplicate.

To further evaluate the analytical sensitivity of the selected sequences, dot blot assays were performed using serial dilutions of recombinant S protein. Both aptamers, seq_2714 and seq_1397, were able to detect the target protein across a range of concentrations. In the serial dilution experiment, 0.013 mg/mL was the lowest RBD concentration tested at which a detectable signal was observed under the assay conditions evaluated (Figure 14). These results indicate that the aptamers maintain a detectable binding capacity at relatively low protein concentrations and confirm their ability to recognize immobilized S protein in a membrane-based assay format.

Dot blot analysis evaluating the recognition profiles of the biotinylated ssDNA aptamers seq_1397 and seq_2714 was conducted. Two recombinant SARS-CoV-2 RBD preparations were evaluated: RBD(1), expressed and purified in-house from a synthetic RBD gene obtained from GenScript, and commercially purified recombinant RBD from Rekon Biotech. Both RBD proteins contained a 6 × His tag. An anti-RBD antibody was included as an independent positive control for RBD detection. His-tagged unrelated proteins, including phi29 DNA polymerase from bacteriophage and NS5 polymerase from West Nile virus, were used as protein controls. Bovine serum albumin (BSA) and a complex protein extract from *Acanthamoeba* were included as unrelated protein and biological matrix controls, respectively. Protein samples were immobilized onto the membrane at a concentration of 0.013 mg/mL and incubated with biotinylated seq_2714 (1 µM) or anti-RBD antibody (0.2 mg/mL). Detection was performed using the streptavidin–HRP system for aptamer binding or the corresponding antibody detection system. Seq_2714 recognized both recombinant RBD preparations, whereas no detectable signal was observed with BSA or *Acanthamoeba* extract.

**Figure 15:**
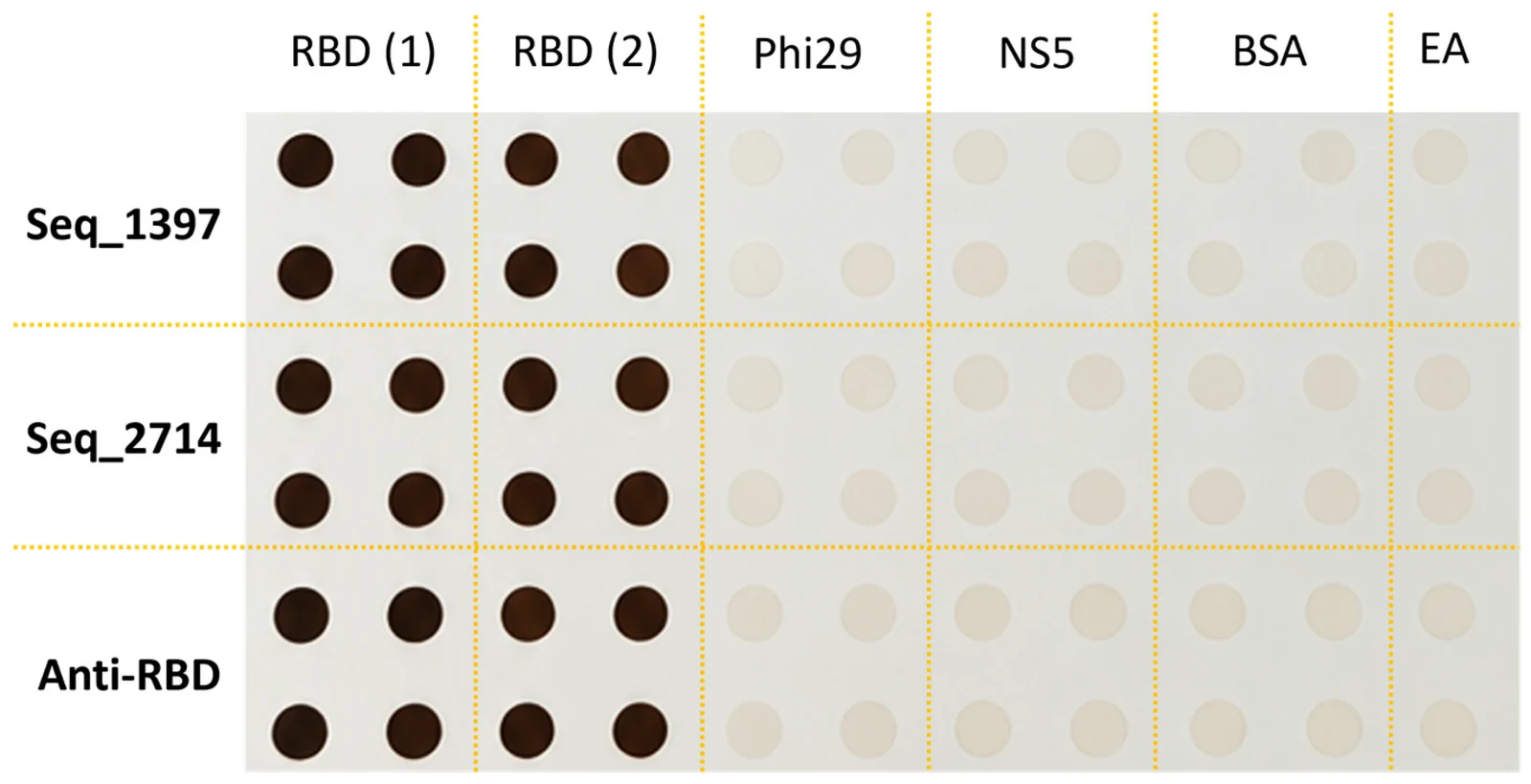
Dot blot analysis of seq_1397 and seq_2714 recognition of the recombinant SARS-CoV-2 RBD and control proteins.

The dot blot analysis showed detectable signals only for the recombinant SARS-CoV-2 RBD samples when detected with seq_2714 or the anti-RBD antibody. No signal was observed with the unrelated protein controls, including His-tagged phi29 polymerase, His-tagged NS5 polymerase, BSA, or the *Acanthamoeba* protein extract, supporting preferential recognition of the recombinant RBD by seq_2714 under the experimental conditions evaluated (Figure 15).

In order to explore the applicability of these aptamers in complex biological matrices, both sequences were subsequently tested against clinical saliva samples and environmental water samples previously analyzed for SARS-CoV-2 presence.

**Figure 16:**
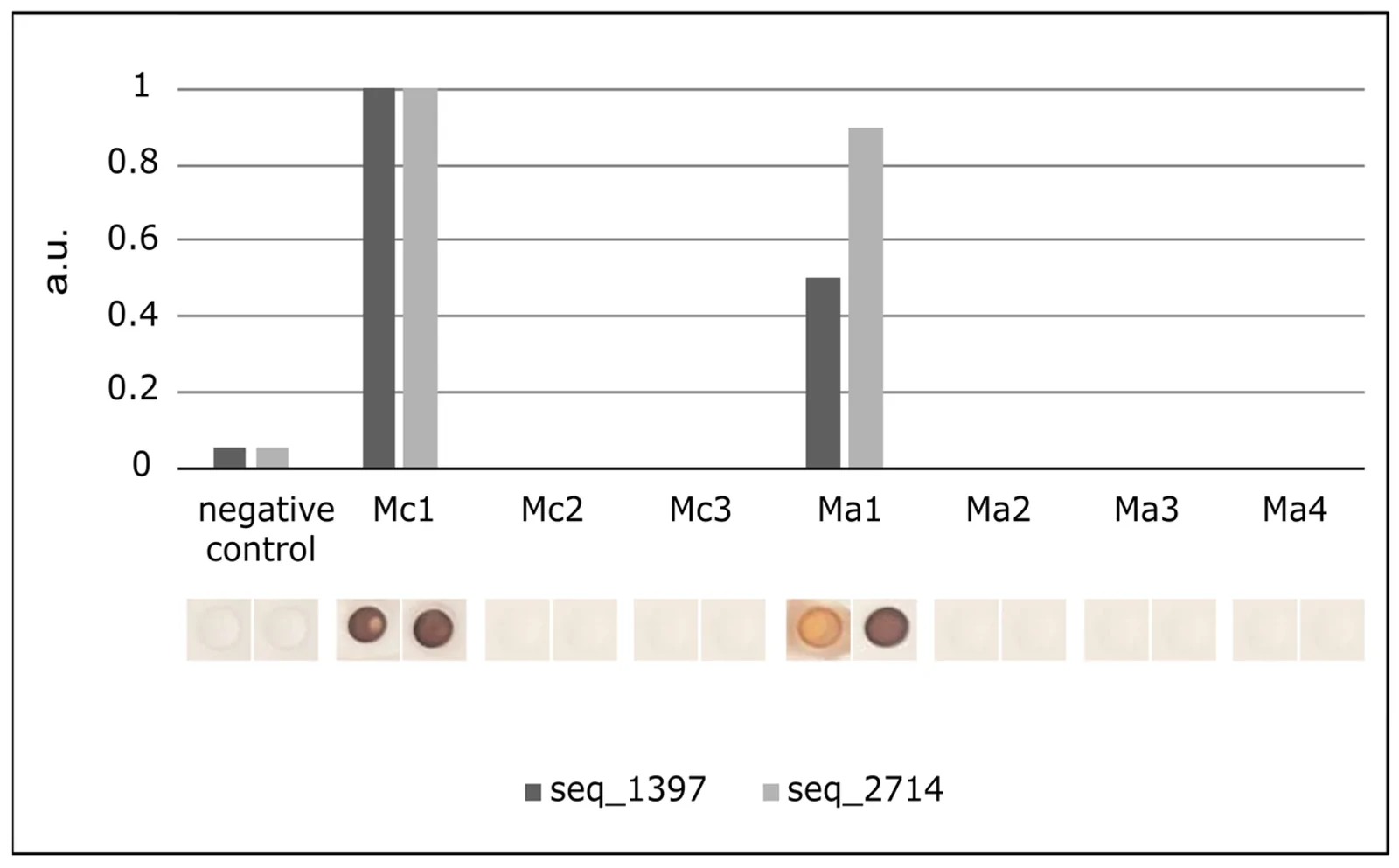
Recognition of the SARS-CoV-2 S protein in clinical and environmental samples using the selected aptamers. Dot blot analysis showing binding of biotinylated aptamers seq_2714 and seq_1397 (1 µM) to proteins present in clinical saliva samples (Mc1–Mc3) and environmental water samples (Ma1–Ma4). Detection was performed using streptavidin–HRP and DAB substrate. Positive signals were observed in one clinical sample and in the environmental sample Ma1, indicating the presence of detectable S protein antigen. Environmental samples Ma2–Ma4 were previously positive for SARS-CoV-2 by RT-qPCR but did not produce detectable signals in the blot assay. Data are presented as the SD of three independent experiments, each performed with triplicate analyses.

Among the three clinical samples analyzed (Mc1–Mc3), a positive signal was detected in one sample using both aptamers, indicating the presence of S protein or Spike-containing viral particles within the sample matrix (Figure 16). Given the limited number of clinical (*n* = 3) and environmental (*n* = 4) samples evaluated, no conclusions regarding diagnostic sensitivity, robustness, or clinical applicability can be established. These analyses were performed as an initial proof-of-principle evaluation to determine whether the selected aptamer could generate detectable signals in complex biological and environmental matrices containing the target antigen.

Environmental samples were also evaluated to assess the performance of the aptamers in water matrices. One environmental sample (Ma1), obtained using a viral particle concentration protocol designed for free-virus detection, showed clear recognition by both aptamers. In contrast, samples Ma2–Ma4, which were previously identified as SARS-CoV-2 positive by RT-qPCR, did not produce detectable signals in the blot assays (Figure 16).

This discrepancy may be explained by differences in the concentration protocols used. Samples Ma2–Ma4 were processed using a method optimized for *Acanthamoeba* detection, which may result in viral particles being internalized within amoebae or associated with cellular structures. Such compartmentalization could potentially mask or limit the accessibility of S protein epitopes, thereby preventing aptamer binding under the assay conditions.

Collectively, these experiments showed recognition of recombinant SARS-CoV-2 RBD by the selected aptamers under the tested blot-based conditions and provided preliminary evidence that detectable signals can also be obtained in selected clinical and environmental samples. Given the limited number of complex samples analyzed, these findings should be considered exploratory and do not establish general analytical or diagnostic performance.

Dot blot assay evaluating the binding of biotinylated ssDNA aptamers seq_1397 and seq_2714 to recombinant SARS-CoV-2 RBD proteins. Two RBD preparations were analyzed: RBD(1), expressed and purified in-house from a GenScript-derived construct, and commercial recombinant RBD from Rekon Biotech (both 6 × His-tagged). Anti-RBD antibody was used as a positive control, while His-tagged phi29 and NS5 polymerases, BSA, and Acanthamoeba protein extract were included as unrelated controls. Protein samples (0.013 mg/mL) were incubated with aptamers (1 µM) or anti-RBD antibody (0.2 mg/mL). Detection was performed using streptavidin–HRP or anti-mouse-HRP.

## 4. Discussion

Recent studies provide an important context for interpreting the present results. Auti et al. (2025) combined conventional SELEX-based aptamer selection with experimental binding assays and subsequent computational docking against the SARS-CoV-2 RBD [8], whereas Poolsup et al. (2025) combined SELEX with quantitative affinity characterization and epitope mapping of S-protein-binding aptamers [7]. In contrast, the present study explores a computational-first strategy in which structural prediction and molecular docking are used at the initial selection stage to prioritize candidate ssDNA sequences before experimental evaluation. Thus, the principal contribution of our approach is not the identification of RBD-binding aptamers itself but the exploration of computational screening as a means of reducing the experimental search space prior to experimental testing.

The present study demonstrates that a computational-first strategy integrating structural modeling, energetic filtering, and molecular docking can effectively streamline the prioritization of candidate DNA aptamers targeting the SARS-CoV-2 S protein for subsequent experimental testing. While conventional SELEX-based approaches remain the gold standard for aptamer selection, they typically require multiple iterative enrichment cycles, extensive wet-lab screening, and significant time investment [36,37]. In contrast, our approach reduced an initial combinatorial space of 4000 sequences to a small subset of three highly stable and energetically favorable candidates prior to synthesis, thereby minimizing experimental attrition and focusing validation efforts on structurally optimized molecules.

Importantly, only a limited number of studies have reported in silico-guided aptamer discovery workflows followed by experimental assessment of computationally prioritized candidates. In the present study, Western blot and dot blot assays were used as an initial experimental assessment of the ability of the selected aptamers to recognize immobilized recombinant RBD under the conditions tested. In addition, exploratory experiments using a small number of clinical and environmental samples were performed to determine whether detectable signals could be obtained in more complex sample matrices. By combining tertiary structure modeling with potential energy analysis and molecular docking against multiple conformational states of the S protein (closed, open, and transitional), we were able to identify candidates showing favorable predicted docking interactions when independently evaluated against closed, open, and transitional S protein conformational models. This is particularly relevant for S protein, whose conformational plasticity directly influences receptor binding and immune evasion.

According to the docking results, seq_2714 was identified as the top-ranked candidate, as it showed favorable predicted docking scores when independently evaluated against the three selected S protein conformational models and exhibited the most favorable average docking score across these models. Although seq_2714 shows its most favorable docking score for the transitional state, and this conformation is more transient than the closed state, targeting this conformation is relevant for interrupting or preventing ACE2 binding. Since the three RBDs must adopt the up conformation prior to interacting with ACE2, early aptamer binding to any of these domains during its conformational transition could interfere with the completion of the conformational changes required for the S protein to become fully competent for ACE2 interaction and subsequent events such as membrane fusion.

The docking energies obtained (up to −10.1 kcal/mol) provided a useful comparative metric for ranking the computationally generated candidates. However, these docking-derived scores should not be interpreted as experimental binding affinities or directly compared with K_D values reported for SELEX-derived aptamers.

**Figure 17:**
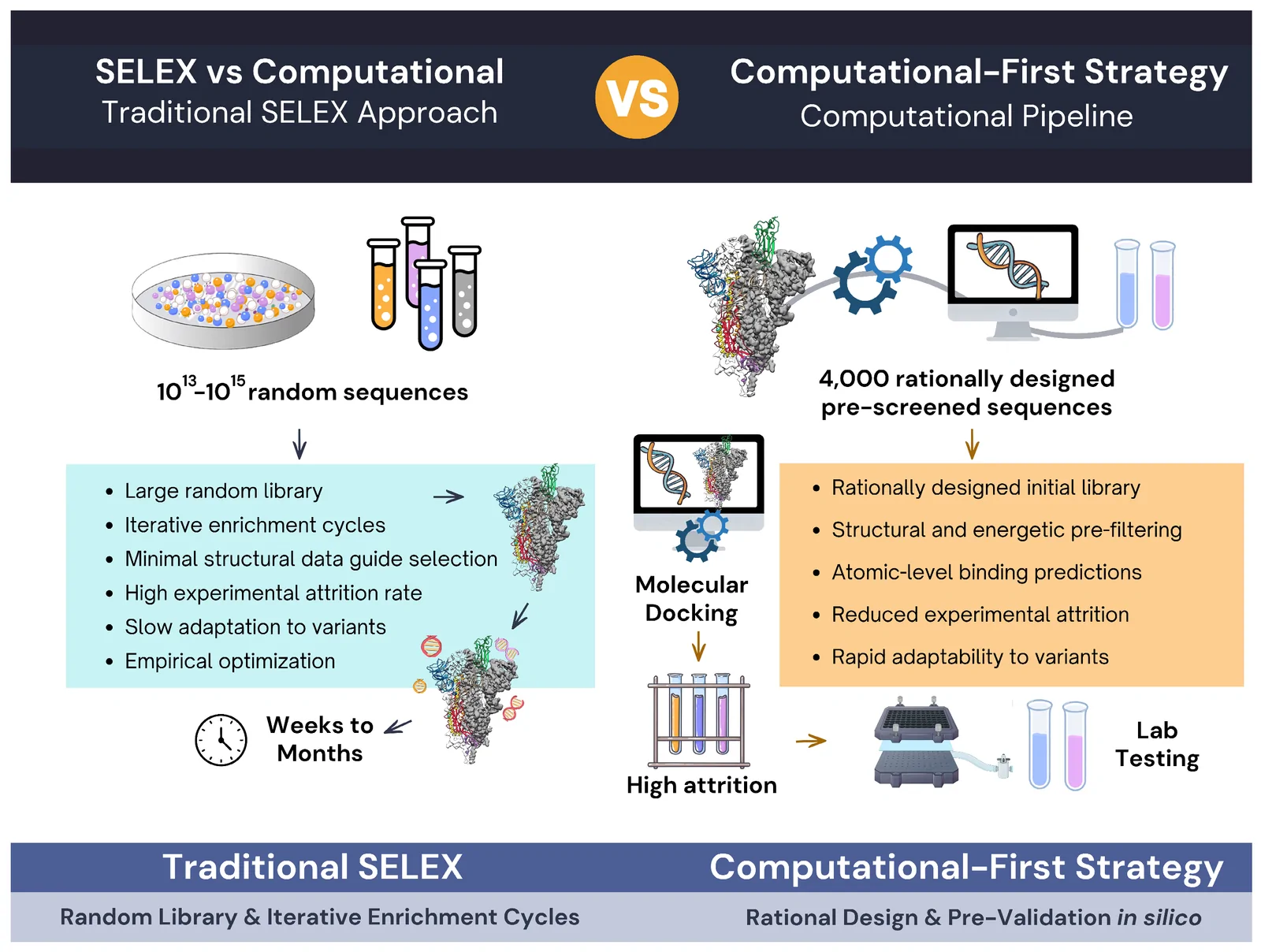
SELEX vs. computational aptamer design. Schematic comparison of the traditional SELEX workflow and computational-first strategies for aptamer discovery, highlighting how in silico structural modeling and docking can reduce the experimental search space and accelerate candidate selection.

The conceptual comparison between traditional SELEX workflows and computational-first strategies further illustrates the methodological advantages of this approach. As shown in Figure 17, SELEX relies on iterative experimental enrichment cycles starting from extremely large random libraries, whereas computational pipelines enable structural modeling, energetic filtering, and molecular docking to prioritize promising candidates prior to experimental testing. This strategy significantly reduces the experimental search space and accelerates the identification of functional aptamers. A further consideration is the different structural contexts used in the computational and experimental stages of this study. Docking was performed using the trimeric S protein to evaluate the predicted aptamer interactions within different S protein conformational states, whereas experimental validation was conducted using isolated recombinant RBD. The localization of several predicted interaction regions within the RBD/RBM provided the rationale for using the isolated recombinant RBD as an initial experimental target. Nevertheless, recognition of the monomeric RBD does not necessarily demonstrate that the same molecular interactions occur within the native trimeric S protein architecture. In particular, interactions involving epitopes formed or stabilized by the quaternary structure of the S protein trimer or by adjacent protomers would not be reproduced using the isolated RBD. Conversely, RBD epitopes accessible in the isolated domain may exhibit different accessibility within the trimeric S protein. Future studies using native-like trimeric Spike will, therefore, be required to determine whether the interactions observed with recombinant RBD are preserved in the complete S protein architecture.

Future studies could further validate these findings by performing additional experiments using native-like trimeric S protein. Ideally, these experiments could be complemented by site-directed mutagenesis of residues located within the predicted aptamer-binding site. Such studies would help determine whether the recognition observed with the isolated RBD is maintained within the structural context of the complete S protein and, importantly, whether the residues identified by molecular docking actually contribute to the aptamer– S protein interaction.

To further contextualize the novelty of the aptamers identified through our computational-first workflow, their main characteristics were compared with representative SARS-CoV-2 S protein/RBD-targeting DNA aptamers previously obtained predominantly through conventional SELEX approaches (Table 2). It should be noted that the seven candidates listed in Table 1 correspond to seven structural models derived from three distinct 20 nt sequences (seq_2714, seq_1423, and seq_1397), since multiple energetically favorable conformations of seq_1423 were retained after structural clustering. A particularly distinctive feature of the candidates identified here is their compact size. In comparison, previously reported RBD-targeting aptamers such as CoV2-RBD-1C and CoV2-RBD-4C comprise 51 and 67 nucleotides, respectively [3], whereas CoV2-6C3 is a 46 nt aptamer obtained following sequence optimization and was subsequently engineered into the high-affinity circular bivalent cb-CoV2-6C3 aptamer [2]. These differences in sequence length, selection strategy, structural architecture, and binding characterization are summarized in Table 2.

Despite their different sequences, structural architectures, and selection strategies, an interesting convergence can be observed at the level of the targeted protein region. The SELEX-derived CoV2-RBD-1C and CoV2-RBD-4C aptamers were reported to recognize the SARS-CoV-2 RBD and were selected using an ACE2-competition strategy designed to favor candidates capable of interfering with the RBD–ACE2 interaction [3]. Similarly, CoV2-6C3 was developed as an RBD-binding aptamer capable of inhibiting the interaction between the viral RBD and ACE2 [2]. In our study, docking analyses likewise predict that the short 20 nt candidates interact with accessible surfaces within or close to the RBD/RBM, including residues associated with ACE2 recognition. In the transitional S protein state, the prioritized structures were positioned near Ser371, Ser375, Ser477, and Tyr505, with some seq_1423 conformations additionally approaching Glu484, whereas analysis of the closed state identified additional residues, including Lys417, Leu452, Gln498, and Asn501. Thus, although the sequences identified by our computational-first strategy are structurally distinct from previously reported SELEX-derived aptamers, both approaches appear to converge toward biologically relevant and accessible surfaces within the receptor-binding region.

More recent studies further support the relevance of integrating experimental aptamer selection with structural and computational characterization. Poolsup et al. identified three S1-targeting DNA aptamers—AptS1-tSP4, AptS1-tSP10, and AptS1-tSP11—with experimentally determined dissociation constants in the nanomolar range (14–59 nM). Importantly, mass spectrometry-based epitope mapping identified the RBD peptide VGGNYNYLYR as a major binding region of AptS1-tSP10, while computational analyses supported interactions with different S protein conformations [7]. This observation is particularly relevant to our approach, in which aptamer selection explicitly considered the open, closed, and transitional conformational states of S protein. Likewise, Auti et al. recently combined conventional SELEX with secondary-structure prediction and molecular docking to identify RBD-targeting ssDNA aptamers and subsequently optimized AptRBD3 into AptRBD3.3 [8]. These recent studies illustrate the increasing integration of experimental selection with computational structural analysis and support the rationale for incorporating target structural information into aptamer development.

Nevertheless, direct quantitative comparison between the affinities reported for previously described SELEX-derived aptamers and the docking results obtained here would not be appropriate. The K_D values summarized in Table 2 represent experimentally determined binding affinities, whereas the docking scores obtained in the present study are computational estimates used to rank candidate structures. Quantitative biophysical characterization of seq_2714, seq_1423, and seq_1397, for example, by surface plasmon resonance or biolayer interferometry, will, therefore, be required to determine their experimental affinities and establish whether their compact 20 nt architecture provides advantages in affinity, specificity, stability, or manufacturability compared with previously reported RBD-targeting aptamers. Consequently, the novelty of the present candidates lies not in identifying the RBD as a new aptamer-binding target but in demonstrating that a computational-first screening strategy can identify structurally compact and previously unreported ssDNA candidates predicted to recognize biologically relevant RBD/RBM surfaces across different conformational states of the S protein.

The experimental assays provide initial support for the ability of the computational workflow to prioritize candidates capable of recognizing recombinant RBD under the tested conditions. Binding of aptamers seq_1397 and seq_2714 to the target protein was also detected under the denaturing conditions used for Western blot analysis. However, this observation does not demonstrate preservation of the native three-dimensional interaction predicted by the computational docking analysis.

The recognition profile of seq_2714 was further evaluated using unrelated protein controls in both Western blot and dot blot assays. In Western blot experiments, seq_2714 showed clear recognition of recombinant SARS-CoV-2 RBD, whereas no detectable signal was observed with BSA, His-tagged phi29 polymerase, or the complex *Acanthamoeba* protein extract. A weak signal was observed with the unrelated His-tagged NS5 polymerase, indicating that some degree of cross-reactivity cannot be completely excluded under these experimental conditions.

In the present study, both aptamers detected an aptamer-reactive band in the recombinant RBD preparation with an apparent molecular weight of approximately 25 kDa by Western blot. This apparent molecular weight was lower than the theoretical molecular weight calculated for the recombinant RBD used in this study (approximately 30.5 kDa without the purification-tag residues and approximately 31.39 kDa including the C-terminal His6 tag). The basis for this electrophoretic discrepancy was not established in the present study; therefore, the apparent migration at approximately 25 kDa should not, by itself, be interpreted as confirmation of RBD identity.

In contrast, in the dot blot assay, seq_2714 recognized both recombinant RBD preparations, while no detectable signal was observed with phi29 polymerase, NS5 polymerase, BSA, or the *Acanthamoeba* protein extract, and anti-RBD antibody was included as an independent positive control and showed recognition of both recombinant RBD preparations.

Taken together, these complementary control experiments support preferential recognition of recombinant RBD by seq_2714 under the conditions evaluated, while also indicating that the recognition profile may be influenced by the experimental assay format. However, because a scrambled or composition-matched ssDNA control was not included, these experiments do not establish sequence-dependent recognition. Additional studies incorporating appropriate control oligonucleotides and quantitative solution-phase binding approaches will be required to further characterize the specificity and molecular determinants of the aptamer–RBD interaction.

Importantly, the Western blot experiments were performed following SDS-PAGE under denaturing conditions. Therefore, detection of an aptamer-reactive band in the recombinant RBD preparation demonstrates recognition under the experimental conditions evaluated but does not establish recognition of the native three-dimensional RBD conformation or preservation of the molecular interaction predicted by docking. Although the dot blot assay avoids SDS-PAGE and the associated denaturing electrophoretic conditions, the recombinant RBD is passively immobilized on a membrane, which may influence protein orientation, accessibility, and conformation. Therefore, the dot blot results similarly provide evidence of recognition of immobilized recombinant RBD under the assay conditions evaluated but should not be interpreted as direct evidence of binding to the native RBD conformation. Importantly, neither assay establishes that aptamer recognition involves the specific residues or interaction surface predicted by molecular docking. Validation of the predicted binding interface will require additional studies using native-like or solution-phase targets, together with approaches such as site-directed mutagenesis of the predicted contact residues.

Accordingly, the residues identified by docking should be regarded as predicted candidate contact residues rather than an experimentally validated binding interface.

Although several SELEX-derived aptamers have reported nanomolar detection limits (e.g., ref. [38]), those platforms often rely on signal amplification systems, nanoparticle enhancement, or electrochemical readouts. In our case, detection was achieved using a relatively simple blot-based format, suggesting that further signal optimization (e.g., integration into electrochemical or fluorescence-based aptasensors) could substantially improve analytical sensitivity. Importantly, the fact that these aptamers were selected primarily through computational screening considerably shortened development timelines, highlighting a trade-off between rapid design and iterative affinity maturation that may be optimized in future hybrid workflows.

The analysis of clinical and environmental samples provides additional translational insight. A detectable signal was observed in one of the three clinical saliva samples evaluated. Although this observation indicates that aptamer-associated detection can occur in a complex biological sample, the limited sample size does not allow for conclusions regarding general performance in biological matrices, diagnostic sensitivity, or clinical applicability. Future studies including larger cohorts and quantitative comparisons with RT-qPCR or antigen-based immunoassays will be necessary to establish diagnostic sensitivity and specificity parameters.

Interestingly, the environmental analysis revealed positive detection in a concentrated water sample processed for free viral particles (Ma1), whereas samples indicated positive by RT-qPCR did not consistently yield aptamer recognition. In the environmental samples, RT-qPCR positivity indicates the presence of viral RNA but does not necessarily imply the presence of intact or accessible Spike/RBD protein antigen. Therefore, discordance between RT-qPCR and aptamer-based detection may reflect differences in the persistence of viral RNA and protein antigens, antigen degradation or reduced accessibility, or matrix-associated effects. Structural shielding or association of viral material with *Acanthamoeba* could also potentially influence antigen accessibility; however, these possibilities were not directly investigated in the present study and should, therefore, be considered hypotheses rather than mechanisms demonstrated by our data. Further studies directly assessing Spike/RBD integrity and accessibility in environmental samples will be required to determine the basis of this discordance [39,40]. If confirmed, this phenomenon would have important implications for environmental surveillance strategies, as nucleic-acid detection does not necessarily correlate with antigen accessibility.

Beyond its potential diagnostic application, structural modeling showed that seq_2714 obtained favorable predicted docking scores when independently evaluated against closed, open, and transitional S protein conformational models. These results identify seq_2714 as a candidate of interest for further investigation across different structural states of Spike; however, the present docking analyses do not demonstrate that the aptamer remains bound during conformational transitions or establishes dynamic, kinetic, or thermodynamic stability. Molecular-dynamics simulations and experimental binding studies using native-like Spike conformations will, therefore, be required to investigate these properties. Moreover, whether seq_2714 could interfere with Spike–ACE2 interactions or exert any functional antiviral effect remains unknown and was not evaluated in the present study. The predicted docking regions provide a structural hypothesis for future investigation rather than evidence of therapeutic activity. ACE2-competition assays, cell-based viral-entry inhibition studies, and appropriate neutralization experiments will be required to determine whether the computationally predicted interactions translate into a measurable functional effect.

In addition to the docking scores, the docking analysis also provided structural insight into the predicted interaction interface between the selected aptamer and the S protein RBD. The top-ranked docking candidate, seq_2714, was consistently positioned in proximity to the receptor-binding region of the RBD across the analyzed S protein conformations. As illustrated in Figure 3, the aptamer adopts a conformation that allows for extensive surface contact with the RBD region, suggesting that the interaction surface partially overlaps with residues involved in ACE2 recognition. In particular, the predicted binding interface appears to involve residues located within or adjacent to the RBM of the S protein, the region directly responsible for host receptor engagement.

Such positioning is particularly relevant from both diagnostic and functional perspectives, as ligands targeting this region could potentially interfere with receptor binding or selectively recognize conformational states associated with viral entry. The predicted interaction interface covers an extended surface of the RBD, suggesting that the aptamer establishes multiple contact points that contribute to binding stability. Although the precise molecular contacts require further structural and biophysical validation, the docking results support the hypothesis that the computationally selected aptamer targets a structurally and functionally relevant region of the S protein.

An additional consideration regarding the potential applicability of the selected aptamers is the sequence variability of the SARS-CoV-2 RBD. Several residues located within or near the predicted aptamer interaction regions, including K417 and E484, correspond to positions that have undergone substitutions in different SARS-CoV-2 variants. Their proximity to the predicted docking regions highlights the importance of considering viral sequence variability in future characterization of these aptamers. However, the present docking analysis does not establish whether substitutions at these or neighboring positions would preserve, reduce, or enhance aptamer recognition, and, therefore, no conclusions regarding variant robustness or vulnerability can be drawn from these data. Experimental evaluation using RBD or Spike proteins from different SARS-CoV-2 variants will be required to determine whether sequence variation within or near the predicted interaction regions affects aptamer recognition.

Given the polyanionic nature of ssDNA, electrostatic interactions may contribute to aptamer–RBD recognition. In our analysis, the RBD showed a moderately higher combined proportion of lysine and arginine residues than the complete S protein (10.7% vs. 7.9%), and residues with diverse physicochemical properties were located in proximity to the predicted aptamer poses. However, these descriptive observations are insufficient to determine the contribution of electrostatic interactions to aptamer binding, since electrostatic recognition depends on the spatial distribution and accessibility of charged residues at the interaction interface, as well as on factors such as ionic strength, solvent conditions, protonation state, and molecular geometry. Therefore, the present analysis should be considered inconclusive regarding the relative contribution of electrostatic interactions. Competition experiments with unrelated nucleic acids, ionic-strength-dependent binding studies, and quantitative biophysical approaches will be required to further characterize the molecular determinants of aptamer–RBD recognition.

Taken together, the results presented in this study support the feasibility of a computationally guided strategy for prioritizing candidate DNA aptamers targeting the SARS-CoV-2 S protein. By integrating sequence library generation, structural modeling, energetic filtering, and molecular docking prior to experimental testing, the proposed workflow substantially reduced the number of candidates requiring experimental assessment. Subsequent Western blot and dot blot assays showed detectable recognition of recombinant RBD by the selected aptamers under the conditions tested. Exploratory analysis of a limited number of clinical and environmental samples further showed that detectable signals could be obtained in selected complex matrices, although these observations do not establish general analytical or diagnostic performance.

Collectively, these findings support the concept that bioinformatic-driven aptamer engineering can function as a scalable and adaptable platform for rapid molecular recognition tool development. Rather than replacing experimental selection, computational modeling may redefine the early stages of aptamer discovery by shifting part of the initial screening effort from random library screening toward computational candidate prioritization followed by targeted experimental characterization. Beyond the specific case of SARS-CoV-2, the strategy described here may facilitate the accelerated design of aptamer-based diagnostic tools adaptable to emerging pathogens and other biomedical targets.

Future work should focus on evaluating performance against emerging viral variants, introducing post-synthetic chemical modifications to enhance nuclease resistance, and integrating these sequences into portable point-of-care detection systems. The framework presented here illustrates how computational prioritization may accelerate the early stages of aptamer discovery and guide subsequent experimental characterization.

## 5. Study Limitations

Several limitations of this study should be acknowledged. First, an limitation of the computational workflow concerns the use of AutoDock Vina for aptamer–protein docking. Vina was selected at the time of workflow development as an accessible and computationally efficient tool for the comparative screening and ranking of a large number of candidate structures. However, its scoring function and search strategy were primarily developed for relatively small ligands and cannot fully capture the extensive conformational flexibility of a 20-nucleotide ssDNA molecule. Accordingly, the docking scores and predicted poses obtained in this study should be interpreted as comparative tools for candidate prioritization rather than as definitive structural models of the aptamer–S protein complexes. Future studies combining protein–nucleic-acid-specific docking approaches with molecular dynamics simulations will be valuable for refining the predicted complexes and evaluating their conformational stability and interactions over time.

The relatively limited size of the initial computational library (4000 sequences) should also be considered when interpreting the present results. This number was intentionally selected to demonstrate the feasibility of the workflow using standard desktop computational resources and does not approach the sequence diversity of conventional experimental SELEX libraries. Consequently, potentially favorable sequence regions outside the explored computational space may not have been sampled. Future implementation of the workflow will enable substantially larger sequence libraries to be explored as computational.

In addition, the analysis of clinical and environmental samples was exploratory and included only three saliva samples and four environmental water samples. Therefore, these results should be considered preliminary and cannot be used to establish diagnostic sensitivity, specificity, robustness, or clinical performance. Validation in substantially larger and well-characterized cohorts, including appropriate positive and negative controls, will be required to determine the diagnostic potential of the selected aptamers.

The analytical sensitivity of the current dot blot format was not formally established in the present study. In the serial dilution experiment, 0.013 mg/mL was the lowest RBD concentration tested at which a detectable signal was observed under the assay conditions evaluated. This concentration corresponds to approximately 414 nM when calculated using the theoretical molecular weight of the 6 × His-tagged recombinant RBD (~31.39 kDa). However, this value should not be interpreted as a formally established analytical limit of detection (LOD), as replicate measurements across the low-concentration range, assessment of blank or negative-control variability, and a predefined statistical decision criterion were not included. Therefore, the present dot blot assay should be regarded as a proof-of-principle experimental assessment, and further analytical validation will be required to establish its sensitivity and potential applicability as a diagnostic platform.

An additional limitation is the absence of a scrambled or composition-matched ssDNA control. Although the unrelated-protein controls support preferential recognition of recombinant RBD under the conditions evaluated, they do not establish that recognition is sequence dependent. Future studies incorporating appropriate negative-control oligonucleotides will, therefore, be required to assess sequence specificity.

Further characterization of aptamer–target interactions using quantitative approaches such as surface plasmon resonance (SPR) or isothermal titration calorimetry (ITC) would allow for determination of binding affinity and kinetic parameters under controlled conditions. In addition, incorporation of the selected aptamers into more sensitive detection platforms, including electrochemical- or fluorescence-based systems with signal amplification, could substantially improve analytical sensitivity. Future studies should, therefore, focus on quantitative affinity characterization, assay optimization, and validation in larger clinical and environmental sample cohorts.

## 6. Conclusions

In this study, we developed and experimentally evaluated a computational-first strategy for the identification of short ssDNA aptamers targeting the SARS-CoV-2 S protein RBD. By integrating sequence generation, structural modeling, energetic filtering, and molecular docking against multiple S protein conformational states, we reduced an initial library of 4000 candidate sequences to a limited number of structurally favorable aptamers for experimental evaluation.

The selected 20-nucleotide aptamers, particularly seq_2714 and seq_1397, demonstrated the ability to recognize recombinant SARS-CoV-2 RBD under the experimental conditions evaluated by Western blot and dot blot assays. These results provide experimental support for the feasibility of computational prioritization as an effective approach to accelerate the early stages of aptamer discovery. Importantly, the present strategy does not aim to replace conventional experimental selection methods but rather to complement them by reducing the initial screening space and focusing experimental efforts on structurally optimized candidates.

These assays support the recognition of the recombinant RBD under the experimental conditions evaluated; however, they do not establish recognition of the native RBD conformation or validate the binding pose, interaction surface, or contact residues predicted by molecular docking.

The detection of signals in selected clinical and environmental samples provides preliminary proof-of-principle evidence that these aptamers can be further explored in complex sample matrices. However, additional studies involving quantitative biophysical characterization, evaluation against emerging SARS-CoV-2 variants, assessment under native protein conditions, and integration into optimized biosensing systems will be required to fully define their analytical performance and translational potential.

Overall, this work supports the feasibility of computationally guided aptamer engineering as a scalable and adaptable framework for the early-stage development of nucleic-acid-based recognition tools against viral targets.

## Figures and Tables

**Table 1:** Docking scores for the aptamer–S protein complexes in different conformational states of the RBD OS, CS and TS. Aptamer models were ranked based on their average docking scores (kcal/mol) across the different RBD conformational states. The PDB code of the target structure used is indicated in parenthesis.

Model	TS (6XM0)	CS (7DWY)	OS (7KMS)	Average
model-2-seq_2714	−10.1	−8.7	−9.5	−9.4
model-2-seq_1423	−9.6	−8.5	−8.5	−8.9
model-3-seq_1423	−8.7	−9.6	−8	−8.8
model-8-seq_1423	−8.3	−8.9	−7.9	−8.4
model-6-seq_1423	−9.5	−7.5	−7.5	−8.2
model-5-seq_1397	−8.1	−8.6	−7.7	−8.1
model-1-seq_1423	−8.4	−8.6	−7.3	−8.1

**Table 2:** Comparative overview of previously reported SARS-CoV-2 RBD-targeting aptamers and computationally identified aptamer candidates from the present study.

Aptamer(s)	Length (nt)	Selection/ Design Strategy	Target	Experimental Binding Affinity/Computational Docking Score	Binding Region/ Structural Characteristics	Reference
**CoV2-RBD-1C**	51	ACE2-competition bead-based SELEX + computational screening	RBD	Experimental K_D = 5.8 ± 0.8 nM	Predicted to interact with the ACE2-binding interface of the RBD	[3]
**CoV2-RBD-4C**	67	ACE2-competition bead-based SELEX + computational screening	RBD	Experimental K_D = 19.9 ± 2.6 nM	Predicted partial overlap with the ACE2-binding interface	
**CoV2-6C3**	46	SELEX followed by sequence truncation/optimization	RBD	Experimental K_D = 44.78 ± 9.97 nM	Two-hairpin structure; blocks RBD–ACE2 interaction	[2]
**cb-CoV2-6C3**	Bivalent circular derivative	Rational engineering of CoV2-6C3	RBD	Experimental K_D = 0.13 nM	Circular bivalent architecture; enhanced stability and RBD–ACE2 inhibition	
**AptS1-tSP4, AptS1-tSP10, AptS1-tSP11**	—	SELEX + BLI characterization	S1 /RBD	Experimental K_D = 14–59 nM	AptS1-tSP10 recognizes the RBD peptide VGGNYNYLYR; stable interactions predicted in open and closed S protein conformations	[7]
**AptRBD3.3**	—	SELEX + structural optimization + molecular docking	RBD	Detection sensitivity: 4 ng RBD; HDOCK score = −361.56	Optimized single/bulged-loop structure; also modeled against S-UK RBD variant	[8]
**seq_2714** **(present study)**	20	Computational-first screening of 4000 ssDNA sequences	S protein/RBD	Mean docking score = −9.4 kcal/mol	Predicted binding close to biologically relevant RBD/RBM residues; favorable binding across open, closed and transitional S protein states	Present study
**seq_1423** **(present study)**	20	Computational-first screening of 4000 ssDNA sequences	S protein/RBD	Mean docking score ≈ −8.1 to −8.9 kcal/mol depending on structural model	Multiple energetically favorable conformations; predicted interactions close to RBD/RBM residues, including E484 in the transitional state	Present study
**seq_1397** **(present study)**	20	Computational-first screening of 4000 ssDNA sequences	S protein/RBD	Mean docking score = −8.1 kcal/mol	Predicted interaction with RBD/RBM; experimentally evaluated by Western blot and dot blot	Present study

## Data Availability

The original contributions presented in this study are included in the article. Further inquiries can be directed to the corresponding author(s).

## Abbreviations

The following abbreviations are used in this manuscript:

ACE2	Angiotensin-Converting Enzyme 2
BSA	Bovine Serum Albumin
CE-SELEX	Capillary Electrophoresis SELEX
Cryo-EM	Cryo-Electron Microscopy
CS	Close State
DAB	3,3′-DiAminoBenzidine
DTT	DiThioThreitol
DWTPs	Drinking Water Treatment Plants
HRP	HorseRadish Peroxidase
OS	Open State
PBS	Phosphate-Buffered Saline
PBS-T	PBS-Tween-20
RBD	Receptor-Binding domain
RBM	Receptor-Binding Motif
S protein	Spike protein
SD	Standard Deviation
SDS-PAGE	Sodium Dodecyl Sulfate–PolyAcrylamide Gel Electrophoresis
SELEX	Systematic Evolution of Ligands by EXponential Enrichment
ssDNA	Single-Strain DNA
TS	Transition State
US EPA	US Environmental Protection Agency
WWTPs	WasteWater Treatment Plants
